# Americans’ multidimensional relationship with science: Is citizen science a path forward?

**DOI:** 10.1371/journal.pone.0357476

**Published:** 2026-09-11

**Authors:** Brooklynn N. Joyner, Lincoln R. Larson, Caren B. Cooper, K.C. Busch, Corey Johnson

**Affiliations:** 1 Department of Parks, Recreation, and Tourism Management, North Carolina State University, Raleigh, North Carolina, United States of America; 2 Department of Forestry and Environmental Resources, North Carolina State University, Raleigh, North Carolina, United States of America; 3 Department of STEM Education, North Carolina State University, Raleigh, North Carolina, United States of America; University of Amsterdam, NETHERLANDS, KINGDOM OF THE

## Abstract

Cultivating positive relationships between the public and scientific communities requires understanding how diverse populations relate to science and what strategies promote favorable outcomes. In 2021, we evaluated Americans’ relationship with science by examining science beliefs (perceived credibility and value of science) and science connections (science interest, self-efficacy, belonging, and identity) using a demographically-diverse online survey of US residents (N = 1854). Most Americans expressed interest in science (60%) and believed science has value to society (68%). However, women and individuals without a graduate degree were less likely to be interested, and Black Americans were less likely to report value of science. Fewer Americans (39%) felt a sense of belonging to science and only 43% believed science was credible. Citizen science participation was a strong positive correlate of science connections and perceived value of science but inversely associated with perceived credibility of science. Approximately 14% of Americans participated in citizen science, and women, Asian, and Hispanic Americans were underrepresented. Citizen science represents a promising pathway for promoting positive views of science, but it needs to reach diverse audiences, including those already experiencing strained relationships with science, to effectively strengthen relations between science and society.

## Introduction

The U.S. public’s relationship with science is as complex and dynamic as the diverse groups of people and cultures that comprise the country. Each time a new crisis emerges, reports of conflict and decline in public interest, engagement, and/or trust in science typically reignite. These concerns are usually followed by questions such as “how do we fix this?” or “how do we use science to help society?” Such questions are difficult to answer considering that large segments of society – particularly those that have been historically minoritized (e.g., women, people of color, the LGBTQ+ community) – continue to feel unwelcome and excluded from science-based professions [1–3] educational experiences [2,4–6] informal learning opportunities (e.g., museums, citizen science) [7–9], and popular culture (e.g., television, literature, media) [10–12]. Furthermore, political polarization of scientific issues divides public opinion on salient environmental and public health challenges, such as how to respond to climate change [13] address wildlife conservation issues [14,15] and mitigate the spread of zoonotic diseases [16]. “Trusting the science” to inform decision-making on these issues incites debate surrounding which science – or *whose* science – one can trust to be “accurate” and “fair” [17–21].

Despite this complexity and conflict, characterizing and understanding the public’s relationship with science is an important endeavor. A strained relationship between science and society has major consequences. For instance, public opposition to federal funding of science in the U.S. has long been associated with lower levels of general knowledge and interest in science [22–24]. Scientific data collection efforts for large-scale biological monitoring rely heavily on community volunteers whose motivations to participate are often linked to their shared interest, identity, or sense of belonging related to the subject of study (e.g., recreational birders participating in bird monitoring projects) [25–27]. Finally, personal beliefs and attitudes towards researchers or policies often influence how communities cooperate, participate, and contribute to the overall success of various environmental [28,29] and public health initiatives [30,31]. This was most recently exemplified through the COVID-19 pandemic, where the apparent disconnect between factual information and personal beliefs and attitudes led mass segments of the American population to question the credibility of emerging science and refuse to comply with policies created to mitigate the spread of a novel, deadly disease [32–34].

To foster a positive relationship with science, researchers need a better conceptualization of how to define and evaluate that relationship. A vast body of literature has explored how people understand, view, and relate to science in various contexts. However, a synthesis of how we might interpret and examine these experiences as components of a more comprehensive, multidimensional relationship with science, and of what factors might influence those relationships, is lacking. Our study attempts to address these two gaps by examining multiple dimensions of the public relationship with science across a diverse sample of the U. S. population. To do this, we first used existing literature to identify key cognitive and affective dimensions of the public relationship to science (e.g., science beliefs and science connections). Second, we assessed the link between these key dimensions and a tool – citizen science – with demonstrated potential to create positive outcomes pertaining to the public relationship with science.

In our manuscript, we refer to *science* as a cultural domain that encompasses the principles and methodologies of Western scientific practice, predominantly rooted in positivist ideals of objectivity and “truth claiming” [35]. Scientists and the institutions for which they work are seen as representatives of broader scientific culture and systems of practice [36]. However, we recognize that most of the public likely understands science from a more mechanistic perspective as a body of factual knowledge and applied practice in specific fields such as chemistry, biology, physics, and geosciences [37]. How people personally understand and define “science” is also likely to vary across a diverse public encompassing people of varying demographic backgrounds (including non-Western origins), experiences, and access to scientific information.

### Science beliefs and science connections

Decades of research in the fields of public understanding of science and science learning have sought to understand what people know about science, what people believe about science, and how people feel about their own abilities to engage with science [23,38–40]. Since the early 1970s, the National Science Board (NSB) has issued biennial *Science and Engineering Indicators* reports that analyze survey data of public attitudes toward science and technology in the United States [2,22]. As a result of this effort, researchers have chronicled over 40 years of demographic representation in STEM education and careers, as well as public attitudes towards science and technology in the American public. Although the structure of the report and survey items have changed over time, measuring scientific literacy has remained a priority in the NSB’s attempt to identify an “attentive public” capable and interested in taking civic action based on their understanding of science and technology issues. Assessment of scientific literacy has typically encompassed three dimensions: knowledge of scientific norms, knowledge of basic scientific content, and attitudes toward organized science [41]. The first two dimensions have been widely studied, with results suggesting that knowledge alone is *not* the strongest predictor of science engagement; instead attitudes toward science appear to have greater influence on participation in science-related activities and civic engagement on issues of scientific debate [23,24,38,42–45]. Attitudes toward science typically encompass both self-described interest in science-related topics or issues and perceptions of the impacts of science on individuals, society, and policy, with the perceived impact of science primarily examined through personal beliefs about the credibility of science and scientists and the perceived value of science to individuals and society [2,22,23,37,38,45,46].

**Fig 1:**
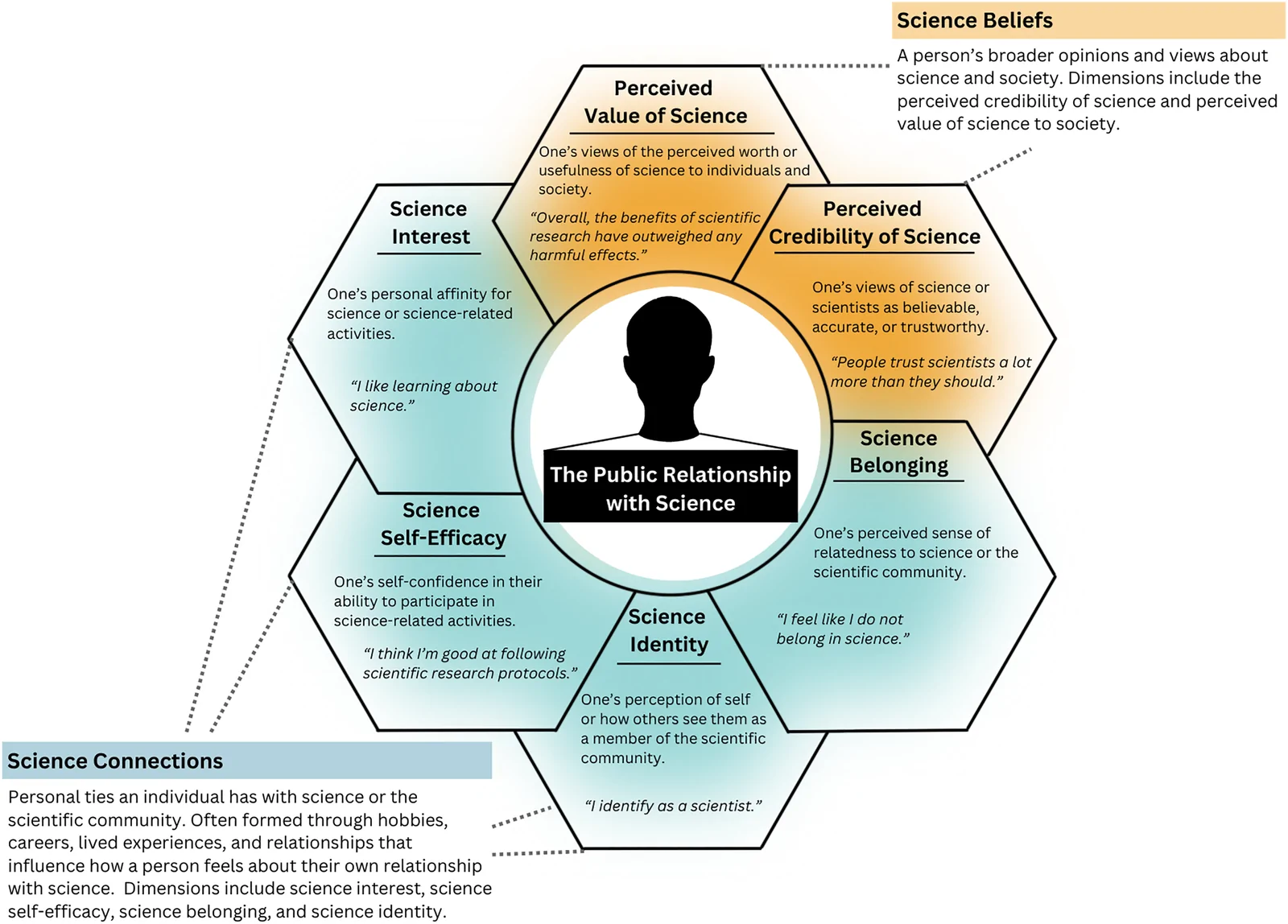
A Multidimensional Relationship with Science: Science Beliefs and Science Connections. The public relationship with science has primarily been examined across six core dimensions that we classify into two broader domains of the relationship – science beliefs and science connections. Science beliefs are broader opinions or views held towards science and include the perceived credibility of science and the perceived value of science. Science connections are typically more affective dimensions that include science interest, science self-efficacy, science identity, and science belonging. Example survey items for each dimension are italicized and described further in the methods section.

While science attitudes are important to assess, individuals’ affective connections to science are also strong predictors of engagement and behavior. Notable work in both formal and informal science learning has demonstrated that individual interest and engagement with science is closely linked to feelings of science belonging, identity, and self-efficacy [40,47–50]. These connections typically emerge through personal ties with science or the scientific community via science-related hobbies, education, careers, interpersonal relationships, and other lived experiences. Although affective connections with science tend to be examined separately from public attitudes towards science [23,38,42], there is evidence to suggest that affect is linked to how people perceive the impact of science to themselves and society. For example, science interest is largely influenced by feelings of competency, belonging, and identity [47,49]. Greater interest in climate science in young adulthood has been associated with perceived credibility of climate science later in life [44]. Our review of the public relationship with science that extends beyond knowledge, particularly one that integrates both personal beliefs and affect manifested through personal connections, revealed six indicators throughout the literature that can be grouped into two larger categories – science beliefs and science connections – all of which are associated with pre-tested instruments designed for evaluation (Fig 1).

Science beliefs consist of the broader opinions or views that individuals hold towards science, such as its *perceived credibility* (views of science as believable, accurate, or trustworthy) and *perceived value* (views of worth or usefulness of science) to society [45,46]. These sets of beliefs can influence other attitudes towards science and affect broader public discourse, behavior, and support for general scientific and technological advancements as well as specific scientific controversies (e.g., climate change) [35,51,52]. Meanwhile, science connections refer to the personal ties that individuals may hold to science or the scientific community, often formed through hobbies, careers, lived experiences, and/or personal relationships that influence feelings of science interest, self-efficacy, identity, and belonging. *Science interest* describes one’s natural affinity or “attentiveness” [41] for science and is indicative of motivations to engage with science [43]. *Science self-efficacy* is one’s perceived self-confidence in their ability to participate in and do science or science-related activities [50,53]. Whether or not someone sees themself, or thinks others recognize them, as a person competent in the skills, culture, and practice of science defines an individual’s sense of *science identity* [40]. Similarly, *science belonging* is one’s own sense of membership in the scientific community and their perceived ‘fit’ as a member with the ‘right’ qualities for that community [40].

Our research aims to build upon the groundwork established by previous studies examining the public relationship with science, while also addressing several shortcomings. First, while science knowledge and scientific literacy have been thoroughly investigated [41,42,54], the role of affective indicators such as self-efficacy or belonging in science have not been adequately explored. Second, it’s rare that more than two or three closely related dimensions (e.g., affective *or* cognitive indicators) are examined simultaneously within a single study. Third, previous studies have not adequately measured and characterized large enough samples from marginalized populations to make meaningful inferences about relationships with science beyond the dominant social groups (e.g., white, educated, urban populations). This continues to perpetuate social inequities by limiting our understanding of why women, people of color, and low-income populations continue to be underrepresented in higher education STEM fields [55,56] and feel excluded from informal science institutions [7,8,57,58]. Given the significant influence that individuals’ science beliefs and science connections have on the public’s participation in science activities and support for science-related policies, building a comprehensive understanding of these domains is critical to fostering a positive public relationship with science. Likewise, it’s important to identify tools and approaches that can cultivate and strengthen people’s beliefs and connections to science. Citizen science represents one potential tool for accomplishing this goal.

### Citizen science as a tool

Citizen science, also referred to as participatory science and community science, is a diverse methodology and discipline that can accommodate various research needs, facilitate broad engagement between public and scientific communities, and help foster positive relationships between science and society by influencing the science beliefs and science connections of individuals that participate [59–62]. Project evaluations from across the citizen science discipline have documented changes following participation in levels of science interest [26,63], science self-efficacy [26,63,64], science belonging [65], and beliefs related to community trust in science [66,67] and the value of science to society [26].

Because citizen science can be tailored to an individual's participation preferences and desired science interests, it offers many viable paths toward science connections across diverse populations [68–70]. For example, thousands of publicly available projects exist across the U.S. to collect data on subjects such as air or water quality [71,72], biodiversity monitoring [61], human health [73], and astronomy [74]. Participation in these projects is also varied and can include a range of tasks that are dependent on the project design such as uploading photos through a mobile device app, collecting and testing household water samples, categorizing data through gamification, participating in organized environmental monitoring surveys, or tracking personal health data. Citizen science can also take the form of an organized grassroots movement where individuals in a community partner with community-engaged researchers to collaboratively define and address environmental or social injustices in their area [75,76]. Considering the broad engagement described above, we define *citizen science participation* as a process where members of public, including non-scientists, participate in one or more of the following scientific activities: identification of issues for study, experimental design, collection of data, data analyses, interpretation of study results, and communication of those results to others. Though the degree of public participation in a project may vary, an emphasis on public interest and impact beyond their participation as research subjects is a defining principle of citizen science [70]. Thus, participation in survey and clinical trial research is often excluded as citizen science practice [77].

### Research aims

In this study, we used a nationally representative survey across multiple racial, gender, age, and socio-economic groups to evaluate the current state of Americans’ relationship with science. Specifically, we addressed these research questions: 1) How do Americans broadly relate to science through science beliefs (e.g., perceived value and credibility of science) and science connections (e.g., science interest, self-efficacy, belonging, identity)?; 2) How do beliefs about science and connections to science vary across different demographic segments of the U.S. population? We also investigated whether a commonly proposed tool for improving relations between science and society – citizen science – might impact these relationships by focusing on these questions; 3) How many Americans have previously participated in citizen science?; 4) How is participation in citizen science associated with Americans’ science beliefs and science connections?

## Materials and methods

We conducted a nationwide web survey across different socio-demographic groups in the United States to better characterize diverse individuals’ relationship with science. Our survey study was nested within a larger project that also aimed to examine public perceptions of citizen science and citizen science participation; For the scope of this paper, we focus on data from this survey that characterizes individuals’ science beliefs, science connections, and citizen science participation.

We administered our survey in two waves using a Qualtrics XM web-based panel of individuals living in the U.S., an increasingly popular technique for large-scale survey research [78]. We supplemented our nationally representative sample with quota sampling for race and ethnicity (at least 20% of responses for White, Hispanic, Black, and Asian communities) to ensure adequate inclusion of historically marginalized populations. This research was approved by the NC State University Institutional Review Board (protocol 14278) prior to data collection and analysis. Respondents were recruited through Qualtrics XM and provided written consent to participate in our study before they were directed to our questionnaire. Responses were collected February 19–27, 2021 (first wave) and October 19–23, 2021 (second wave). After data quality checks and quotas were confirmed, the survey distribution yielded an effective sample size of 1854 U.S. residents.

### Survey instrument

Our survey instrument was designed to broadly understand patterns of science beliefs and connections and to compare those variables among individuals that either have or have not previously participated in citizen science activities. Because our survey asked people to reflect on their personal relationship with science, we did not include a formal definition of “science” that could potentially limit how respondents interpreted and answered survey items about their science beliefs and connections. This allowed respondents to describe personal actions, feelings, and beliefs about science according to their own terms.

To measure science beliefs, we adapted items from Pardo and Calvo’s [45] examination of public attitudes towards science, based on the Eurobarometer, to assess perceived value of science (3 items). We adapted items from Hartman et al.’s [46] Credibility of Science Scale to measure perceived credibility of science (3 items). To measure science connections, our science interest (3 items) and science self-efficacy (3 items) scales were adapted from DEVISE questionnaires [79,80], with adaptations inspired by prior studies examining science interest [53] and self-efficacy [50]. We followed recommendations from Trujillo and Tanner [40] in creating scales that measured science belonging (2 items) and science identity (1 item). For science identity, we reviewed studies examining science and STEM identities (e.g., McDonald et al., Stets et al., [81,82]) and followed van Eeden et al. [83] by using a direct single item measure of subjective science identity: “Do you identify as a scientist?”, allowing a response of “yes”, “no”, or “not sure”. Apart from the single-item measure of science identity, all other statements were rated on a scale from “strongly disagree” (−2) to “strongly agree” (2). Example items for each measure can be found in Fig 1. A full list of items and our complete survey can be found in supporting information S1–S2 Tables, and S1 Survey I nstrument.

We also asked respondents to share their previous experiences with citizen science activities. We began with an introductory statement that broadly defined public participation in science (i.e., citizen science):

Around the world, more and more people are participating in science, whether they are a scientist or not. Here are some of the activities where public involvement in science is growing: identifying issues for study, designing experiments, collecting information or data, conducting analyses, interpreting study results, and communicating those results to others.

We asked respondents to answer two items assessing their previous experience with citizen science. The first item asked “Select ONE response that best describes your involvement in the activities described above”, allowing a singular response from the following options: I have NOT engaged in these types of activities, I am NOT SURE if I have engaged in these types of activities, I have engaged as a leader or coordinator of participants in these activities, I have engaged as a participant in these activities, I have both led/coordinated AND participated in these activities. Following this, respondents were also asked “Which of the following types of scientific research projects have you been involved in?” and allowed respondents to select all applicable responses (Behavioral and social science projects, Environmental and life science projects, Engineering and earth/physical science projects, Health and medical science projects, Other types of projects (please specify)).

Finally, we asked respondents to provide socio-demographic information that included gender, race/ethnicity, age in years, and education. Additionally, we asked respondents to describe their primary occupation or professional occupation as either STEM field or profession (science, technology, engineering, or math) or non-STEM field or profession.

### Analyses

We used exploratory factor analysis to examine the dimensionality of scales measuring latent constructs such as science beliefs and connections. We followed recommendations from Howard [84] to test factorability using the Kaiser-Meyer-Olkin (KMO) Measure of Sampling Adequacy, following this with Principal Axis Factoring (PAF) with an oblique (Promax) rotation that allows for potential correlations among factors. Items with factor loadings of 0.50 or higher were retained, as well as factors with Cronbach alpha values greater than 0.60 and Eigenvalues greater than one. All reverse-coded items failed to meet this threshold and were removed from future analyses. Items that cross-loaded, such as science interest and science self-efficacy items, were merged into a single scale. Items that formed distinct dimensions were aggregated into mean index scores within the original response scales. See supporting information S1–S2 Tables for factor analysis results.

To describe science beliefs and science connections present within the American public, we used descriptive statistics of respondents’ scores for all constructs of interest. Estimates were weighted by race/ethnicity to account for our quota sampling approach and ensure that our response distribution aligned with demographic ratios reported in the US 2020 Census [85]. Following descriptive analysis, we ran a series of unweighted linear or logistic regression models (depending on the distribution of the outcome variables) to explore demographic differences in science beliefs and connections and the associations of citizen science participation with these outcomes of interest. To characterize citizen science participation within the American public, we used descriptive statistics to determine the amount of people participating in citizen science, the modes of participation (participant and/or manager), and the projects’ field of study. This information allowed us to classify respondents into one of three citizen science participation categories: no participation, single-project participant, or multi-project participant. We used a multinomial logistic regression to examine links between socio-demographic variables and the three types of citizen science participation.

## Results

### Science beliefs and connections

Our sampling approach yielded a demographically diverse sample (N = 1854) of US respondents. Overall, 25.5% of respondents identified as White, 20.2% of respondents identified as Hispanic, 20.6% of respondents identified as Black, 21.1% identified as Asian, 4.9% identified as American Indian, and 7.8% identified as multi-racial or “other,” allowing us to adequately characterize perspectives of historically underrepresented racial and ethnic groups not captured in most large-scale quantitative studies of the public relationship with science. In terms of gender, 62.4% of respondents were women, 36.1% were men, and 1.5% were non-binary. The majority of respondents’ ages ranged between 18–34 yrs (48.8%), followed by 35–49 yrs (31.2%), and 50–65 + yrs (20.1%). Overall, 38.8% reported involvement in STEM-related professions. With respect to education, 45.3% did not have a college or graduate degree, 39.8% had a college degree, and 15.0% had a graduate degree. A vast majority of the respondents (87.1%) reported living in urban areas.

**Fig 2:**
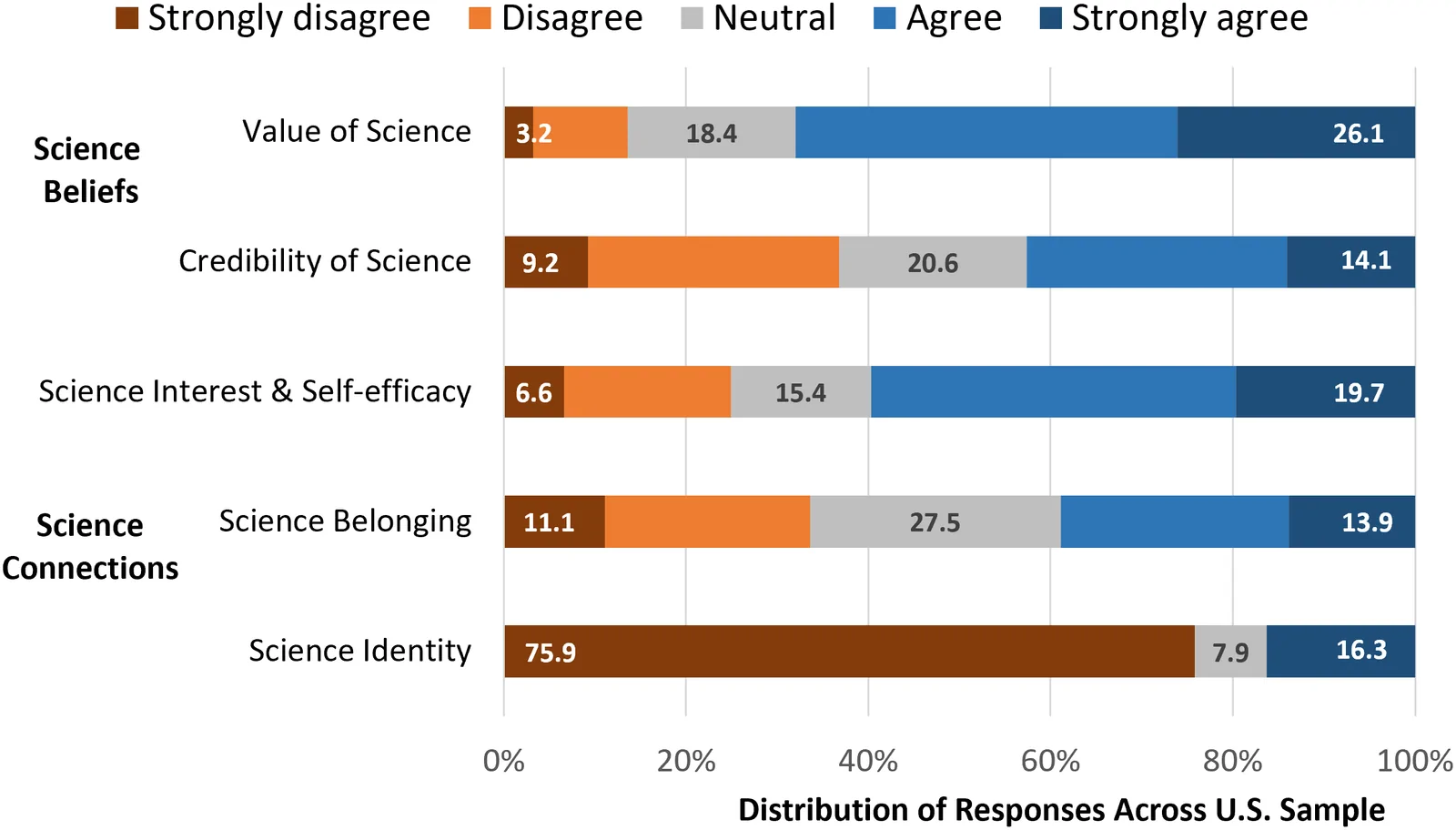
Self-reported Index Scores for Science Beliefs and Connections Among the US Public. Scores are weighted by race/ethnicity to align with demographic ratios in the 2020 US Census. Percentages represent the ratio of respondents who reported scores on each multi-item index according to response options ranging from −2 = Strongly disagree to +2 = Strong agree. The single-item measure of science identity allowed for three responses: yes (coded as strongly agree), unsure (coded as neutral), no (coded as strongly disagree).

After weighting the sample by race/ethnicity, according to self-reported survey data measuring public beliefs about science, 68.3% of Americans agree that science has value to society (M = 0.60, SD = 0.84), with 26.1% reporting strong agreement (Fig 2). Comparatively, 42.6% of Americans reported agreement regarding the credibility of science (M = 0.10, SD = 0.94), while 36.8% of individuals disagreed. In terms of self-reported connections with science, 60.5% of Americans agree, including 19.7% who strongly agree, that they are either interested in science or feel confident that they can “do” science (M = 0.37, SD = 0.88). Overall, approximately 38.9% of Americans reported feelings of belonging with the scientific community (M = 0.12, SD = 1.04), though most (83.8%) do not explicitly identify as a scientist (M = 0.17, SD = 0.40).

**Fig 3:**
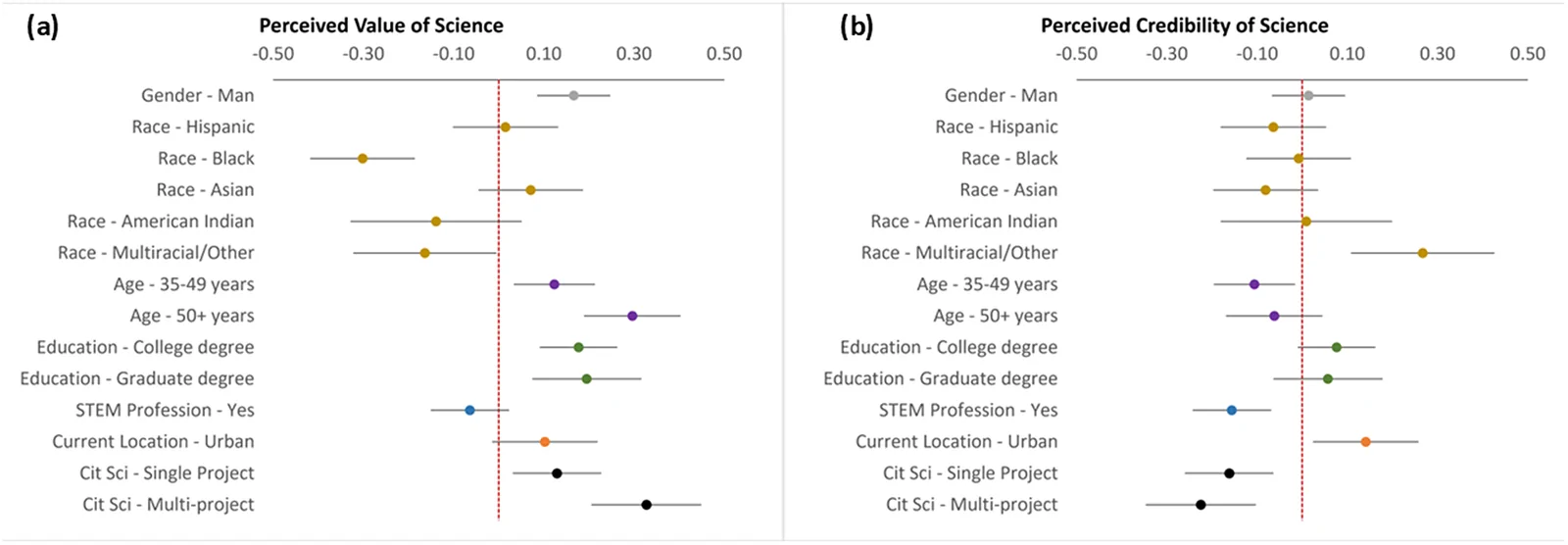
Demographic Predictors of Science Beliefs: Perceived Credibility of Science (a), and Perceived Value of Science (b). Forest plots represent unstandardized (B) parameter estimates (95% CI) for demographic variables in linear regression model predicting outcomes of perceived credibility and perceived value of science among U.S. respondents (n = 1,297). Reference categories: race = White, age = 18-34 years, education = high school, citizen science participation = none.

Our linear regression model comparing science beliefs across demographic groups yielded relatively weak predictive power (Adj. R^2^ < 0.10) but highlighted several key correlates (parameter estimates in S3 Table). Individuals were significantly more likely to report that science has value to society (Fig 3a) if they were male (β = 0.10, p < 0.001), either ages 35−49 (β = 0.07, p = 0.016) or over 50 (β = 0.14, p < 0.001), or had a college (β = 0.11, p < 0.001) or graduate degree (β = 0.08, p = 0.006). While there were not significant differences among most racial/ethnic groups, Black Americans were significantly *less* likely to report that science has value to society (β = −0.149, p < 0.001) compared to White Americans. Regarding views towards the credibility of science (Fig 3b), STEM occupation was the strongest correlate of credibility, with STEM professionals significantly less likely to view science as credible (β = −0.08, p = 0.008). The only other significant correlate of science credibility was Multiracial or Other race, with individuals in that group more likely to view science as credible (β = 0.08, p = 0.019).

**Fig 4:**
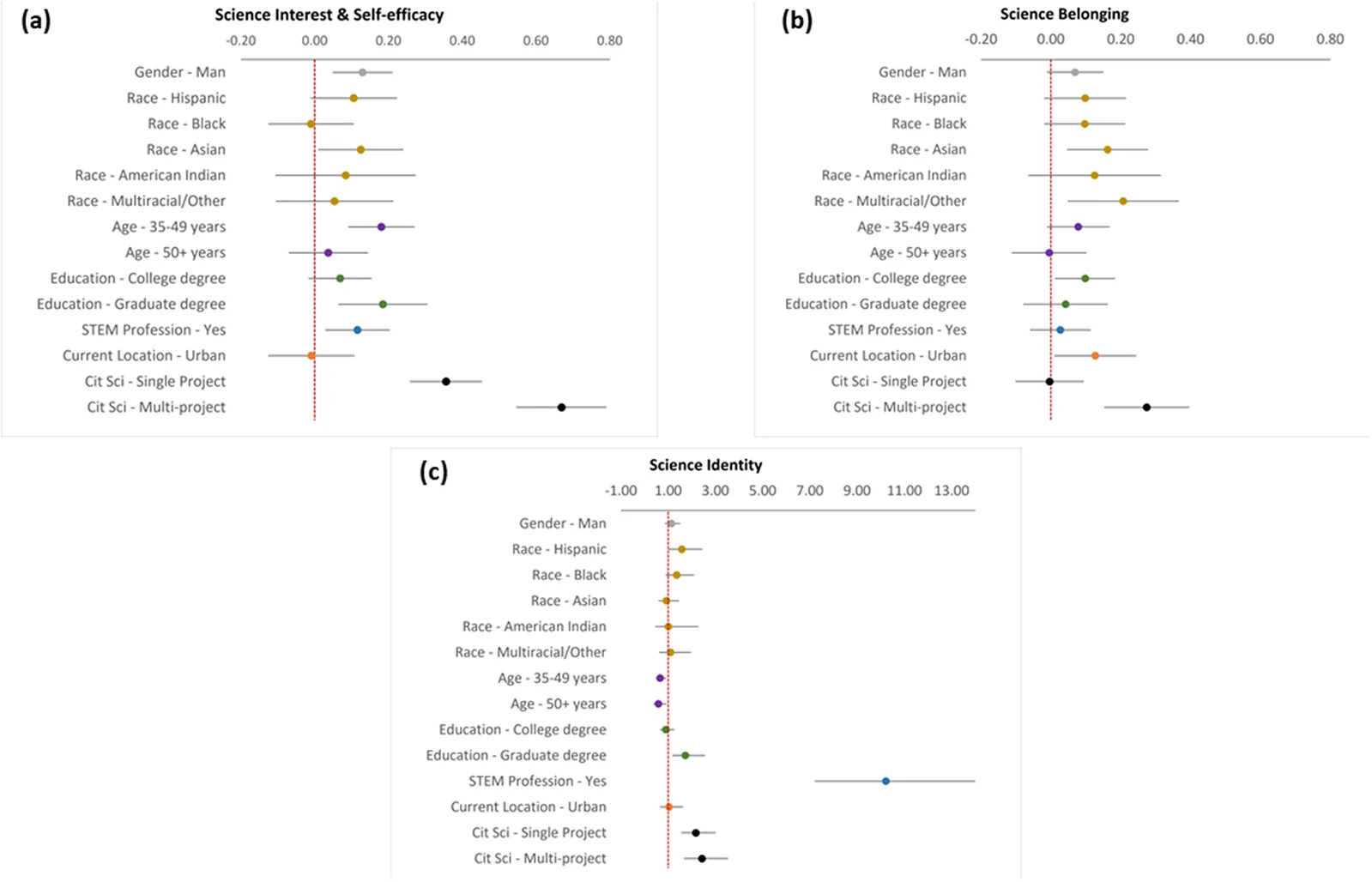
Demographic Predictors of Science Connections: Science interest, Self-efficacy (a), Science Belonging (b), Science Identity (c). Forest plots represent unstandardized (B) parameter estimates (a,b) or odds ratios (c), with 95% CI, for variables in linear (a,b) or logistic (c) regression models predicting outcomes among U.S. respondents (n = 1807) for three dimensions of science connections – (a) science interest and self-efficacy, (b) science belonging, and (c) science identity. Reference categories: race = White, age = 18-34 years, education = high school, citizen science participation = none.

Our linear regression model comparing science connections across demographic groups also yielded relatively weak predictive power (Adj. R^2^ < 0.12) but revealed several key correlates (parameter estimates in S4 Table). Individuals were significantly more likely to report high levels of science interest and self-efficacy (Fig 4a) if they were male (β = 0.07, p = 0.002), Asian (β = 0.06, p = 0.034), ages 35–49 (β = 0.10, p < 0.001), had a graduate degree (β = 0.08, p = 0.003), or worked in a STEM profession (β = 0.06, p = 0.009). Only race/ethnicity was a significant correlate of science belonging (Fig 4b), with respondents who were Asian (β = 0.06, p = 0.029) and Multiracial/Other (β = 0.05, p = 0.042) more likely to report stronger connections.

Our logistic regression model examining demographic correlates of science identity yielded stronger predictive power (Nagelkerke R^2^ = 0.34) and some similar patterns (Fig 4c, parameter estimates in S5 Table). Notably, individuals with a graduate degree (OR = 1.73, p = 0.006), and those who worked in STEM professions (OR = 10.22, p < 0.001) were significantly more likely to identify as a scientist. Hispanic respondents were also slightly more likely to identify as a scientist (OR = 1.59, p = 0.041). Conversely, science identity was lower for older individuals in the 35–49 (OR = 0.66, p = 0.013) and over 50 age groups (OR = 0.59, p = 0.023).

### Citizen science participation

Based on weighted estimates, measurements of public participation in citizen science revealed that the majority of Americans (63.7%) have not engaged with citizen science activities in any form, while 36.3% said they had participated in some capacity. When asked about projects spanning disciplines, 22.3% of respondents said they had engaged in a single project (within one discipline) while 14.0% said they had engaged in multiple projects (across disciplines). When examining participation in more detail, 15.5% of the public participated in behavioral/social science projects, 15.4% in environmental and life science projects, 12.8% in health/medical science projects, and 12.2% in engineering and earth/physical science projects. Based on open-ended responses, many individuals who reported single project participation believed our survey counted as citizen science engagement. As survey research is not considered citizen science engagement [67], we believe our more conservative estimate of multi-project participants (14%) is likely closer to the true proportion of citizen science participants in the American public.

Our multinomial regression model examining demographic correlates of citizen science participation yielded moderate predictive power (Nagelkerke R^2^ = 0.17) and revealed several key relationships (Table 1). Single-project participants were less likely to be Asian (OR = 0.49, p < 0.001) or over age 50 (OR = 0.63, p = 0.010); they were more likely to be men (OR = 1.38, p = 0.010), have a college (OR = 1.37, p = 0.022) or graduate degree (OR = 1.81, p = 0.001), or work in a STEM profession (OR = 3.22, p < 0.001). Patterns were similar for multi-project participants. Individuals engaged in multiple projects were less likely to be Asian (OR = 0.42, p < 0.001) or Hispanic (OR = 0.57, p = 0.018) or over age 50 (OR = 0.41, p < 0.001); they were more likely to be men (OR = 1.49, p = 0.011), have a college (OR = 1.92, p < 0.001) or graduate degree (OR = 2.82, p < 0.001), or work in a STEM profession (OR = 4.31, p < 0.001).

**Table 1:** Demographic Predictors for Citizen Science Participation Among U.S. Respondents.

		Single-project Participation			Multi-project Participation		
Variables	Mean	B	SE	OR	B	SE	OR
**Gender (man)**	0.36	0.323	0.125	1.38*	0.396	0.155	1.49*
**Race** **(ref. = White)**	0.26						
Hispanic	0.20	−0.053	0.183	0.95	−0.557	0.236	0.57*
Black	0.20	0.141	0.180	1.15	−0.269	0.223	0.76
Asian	0.21	−0.718	0.194	0.49***	−0.859	0.229	0.42***
American Indian	0.05	0.395	0.291	1.48	0.207	0.366	1.23
Multiracial/Other	0.08	−0.202	0.255	0.82	−0.316	0.302	0.73
**Age** **(ref. = 18–34 years)**	0.49						
35-49 years	0.31	−0.042	0.140	0.96	−0.224	0.174	0.80
50 + years	0.20	−0.461	0.178	0.63**	−0.882	0.242	0.41***
**Education** **(ref. = high school)**	0.45						
College degree	0.40	0.313	0.136	1.37*	0.654	0.176	1.92***
Graduate degree	0.15	0.597	0.185	1.81***	1.035	0.221	2.82***
**STEM profession (yes)**	0.39	1.167	0.128	3.22***	1.459	0.162	4.31***
**Current location (urban)**	0.87	−0.039	0.188	0.96	−0.345	0.217	0.71

Unstandardized beta parameter estimates (B), standard errors (SE), and odds ratios (OR) for variables in the multinomial regression model predicting single-project and multi-project citizen science participation (relative to the reference category of no participation) among US respondents (n = 1,807). *, **, *** denote statistically significant Odds Ratio (OR) at α = 0.05, 0.01, and 0.001, respectively. Unweighted percentage of participants reporting single-project participation = 22.4%, multi-project participation = 13.1% (with no citizen science participation as the reference category). Model Fit Statistics: n = 1,807; Cragg-Uhler (Nagelkerke) *R*^*2*^: 0.173, χ^2^(558) = 616.6, *p* < 0.001.

### Links between citizen science and science beliefs and connections

Regression models revealed strong links between citizen science participation and both science beliefs (Fig 3 and S3 Table) and science connections (Fig 4, S4 and S5 Tables). Compared to non-participants in citizen science, single-project participants (β = 0.06, p = 0.022) and especially multi-project participants (β = 0.13, p < 0.001) were significantly more likely to value science. However, both single (β = −0.07, p = 0.015) and multi-project participants (β = −0.08, p = 0.007) were less likely to view science as credible. Science connections were strong among citizen science participants. For example, single-project (β = 0.17, p < 0.001) and multi-project participants (β = 0.26, p < 0.001) reported higher levels of science interest and self-efficacy, and multi-project participants reported a stronger sense of science belonging (β = 0.09, p < 0.001). Science identity was also higher among single (OR = 2.17, p < 0.001) and multi-project participants (OR = 2.43, p < 0.001) compared to individuals who did not engage in citizen science. Among all of the potential correlates that we studied, citizen science participation was consistently among the stronger predictors of science beliefs and connections (Figs 3 and 4).

## Discussion

Our results revealed the complexity and diversity of the public’s relationship with science. Overall, most Americans believe science has value to society. However, there are large segments of the public, such as communities of color and women, who could feel alienated from science and its benefits. Furthermore, our results suggest that participation in citizen science might help to facilitate or strengthen those relationships. Below, we examine the meaning and implications of the broader public’s relationship with science and then consider the potential impact of citizen science on this relationship.

### Americans’ relationship with science

Overall, most Americans are interested in science (~60%) and believe that science has value to society (~68%). For the most part, these patterns hold across diverse demographic groups. Our results are comparable to the five decades of stable data collected by the National Science Board, where close to 70% of the U.S. population reported general interest in science [23,86] and 80–89% reported strong beliefs in the value of science in society [2,23,86,87].

Also consistent with previous research, we found that individuals without a college degree and Black Americans are significantly less likely to believe that science has value to society. Other studies support the notion that those who are more familiar with or knowledgeable about science and scientists, perhaps through work or education, usually hold more positive beliefs about science in general [2,42,88,89]. This could explain why individuals in our study who attended college (where some science coursework is the norm) reported more positive beliefs about science’s value to society. However, scholars agree that science knowledge alone is not enough to shape the complex beliefs people hold about science [38,42] and lived experiences related to personal, political, and religious identities or values can strongly influence how some people connect with science and what they believe about it [7,90,91]. This includes experiences with science shaped by historic marginalization and exploitation of certain groups. Consequently, Black Americans may not believe that the practice and benefits of science fully extend to communities of color [35,92] and women may report less interest or confidence in practicing science compared to men [93,94]. The trends we observed are likely an artifact of historic exclusion of these groups from science but also underscore the present need to challenge systemic inequities that persist in science spaces.

In our study, compared to older Americans, adults ages 18–34 were significantly less likely to believe that science has value to society. This appears to contrast a study conducted by Besley et al. [95] where younger adults were more likely to hold positive views of science. Whether or not this is a consistent pattern is unknown, as most public opinion research on science beliefs and attitudes has not accounted for age differences. One possible explanation for our results could be the greater extent to which younger generations engage with science and science news over the internet and social media [96] and the potential influence of those social media sources in shaping people’s perceptions, beliefs, or behavior regarding social or environmental issues [97–99]. Although social media can be an effective tool for engaging younger and broader audiences with science [96,100] the circulation of misinformation and divisive politicization of science over social media is also widespread [101–103]. Future research should explore to what extent, and how, social media engagement and other factors influence young adults’ science beliefs and science connections.

Aside from the groups described above, our data suggest most Americans agree that the benefits of science to society outweigh the negative impacts, and that science makes people’s lives healthier, easier, and more comfortable. But our findings also suggest only ~43% of Americans are inclined to believe science is credible. This number is comparable to Pew Research Center and USGS reports of public confidence in science for the last twenty years [104], which has been largely stable until a slight decline beginning in 2018 [105]. Though our model and predictors for understanding the perceived credibility of science could be improved, it is worth noting these concerns were particularly prominent for individuals who occupied a STEM profession or participated in citizen science. In other words, individuals who presumably had more formal training or familiarity with science were less likely to see it as credible. Previous research has typically found a positive relationship between education and/or familiarity with science (e.g., science undergraduates, science professions) and perceived trustworthiness of science [35,37,88,106]. But there is also research suggesting scientists apply a higher level of scrutiny or skepticism towards scientific work [107], perhaps increasing the threshold for perceived credibility for these individuals. Greater skepticism towards an institution does not necessarily equate to lower levels of trust in that institution [108]. It may simply be that individuals who have directly experienced the scientific process evaluate the credibility of science with a more critical lens relative to nonscientists. More research is necessary to explore the relationship between science skepticism and factors that shape scientists’ relationship to science.

Substantial qualitative and quantitative work to date has examined people’s sense of belonging [47,50] and could help to promote Asian Americans’ sense of belonging in science. These findings underscore the importance of improving equity and inclusivity in science and highlight a need to increase the public-facing visibility of individuals in science with marginalized identities.

### Citizen science in building the public’s relationship with science

Our study found that in 2021, approximately 14% of the American public had engaged with citizen science in some capacity. This estimate is similar to the 10% reported by Pew Research Center in 2020 [109], one of the few other national estimates for citizen science participation of which we are aware. Moreover, citizen science participation was among the strongest predictors of positive outcomes for several dimensions of our defined public relationship with science. Relative to non-participants, those who participated in citizen science were significantly more likely to be interested in science, feel confident in their abilities to do science, feel a sense of belonging in science, identify as a scientist, and believe that science has value to society. Citizen science participation was a better predictor than STEM occupation for science interest and self-efficacy, the perceived value of science, and sense of belonging in science, suggesting there may be a unique link between these outcomes and participating in science as a leisure activity, compared to a profession. While the relationship between citizen science participation and these positive outcomes is clear, our study design could not discern if citizen science facilitates these positive changes or simply attracts participants who already hold more positive beliefs and connections with science. Citizen science engagement is voluntary and self-selecting by nature, with participants primarily motivated to join through personal interest, the desire to learn, and the opportunity to contribute to scientific research or conservation efforts [27,110,111]. Nonetheless, previous research indicates citizen science participation can positively strengthen and reinforce how people think and feel about science [26,63,67]. In essence, evidence suggests that engaging more people in citizen science does help foster a positive relationship with science.

The unexpected inverse relationship between citizen science participation and the perceived credibility of science and scientists merits further inquiry. Citizen science participation was linked to weaker beliefs about the credibility of science, and sustained participation in citizen science appeared to solidify that belief. Other research shows that citizen science can build public trust in science by involving the public more directly in scientific research, thus enhancing access, transparency, and understanding of the scientific process [66,67,112]. How might our surprising results be explained? In theory, citizen science should be capable of confronting misconceptions and “recalibrating” public expectations of how research is conducted, how science is communicated to the public, and who can train to be a scientist. However, public reactions to “real-time” science during the COVID-19 pandemic were a reminder that sudden exposure to the uncertainty and nuance of science can have unexpected consequences, potentially increasing skepticism of science itself [31]. Within the context of citizen science, projects insert nonscientists directly into the scientific process, where misconceptions about objectivity and the determinate nature of science can be challenged. If participants’ expectations of “legitimate” science are unmet, it could result in them perceiving science as less credible. This negative outcome could potentially be avoided if projects are able to address mismatched expectations upfront and help participants reframe new experiences with science. However, most citizen science projects are primarily designed to achieve research goals, not direct transformative learning in participants [113], meaning project leaders are likely not aware or not equipped to challenge participant beliefs about the credibility of science. To better understand the influence of citizen science on the public’s perceived credibility of science or trust in science, more research is needed to explore these constructs as potential outcomes of citizen science participation. This includes improving our conceptual understanding of public trust in science, as well as the methods and tools we use to evaluate it. For example, public engagement with the idealized concept of science (perhaps through media consumption) might differ from direct engagement with science in practice.

Although citizen science has the ability to strengthen science beliefs and connections, there is still a need to improve accessibility and inclusivity in public engagement for citizen science to create widespread change. Our study’s more expansive definition of citizen science (which included most forms of participatory science) is likely to have captured a greater variety of projects and number of participants than most previous work on the topic. This may have contributed to our sample of citizen science participants being more diverse than expected, with only Asians and Hispanics less likely to report participation than White respondents. Even so, participants were still more likely to be highly educated, male, or in a STEM profession, which is consistent with previous measures of volunteer demographics in participatory sciences [9,58,114], underscoring concerns about inclusivity. If citizen science is to be a tool for improving relations between science and society, more work has to be done to understand and engage communities that may already have a strained relationship with science and are thus unlikely to volunteer to participate [115].

### Limitations and future research

Limitations of our study included the potential for sample and response biases to the online self-reported survey, as well as challenges related to measuring abstract constructs and concepts at a large scale. Self-reported data introduce the possibility for social desirability or acquiescence bias when responding to survey items, particularly for items that may elicit a fear of judgment from respondents [116] such as those measuring perceptions of the credibility or value of science during a politically polarizing pandemic. Respondents’ science identity and professional STEM affiliations are also subjective and more complex than our single-item measures could capture. As our study showed, for instance, STEM affiliation does not necessarily indicate science identity. Our goal of evaluating multiple latent constructs in a single questionnaire limited our assessment to just a few items per construct, making it necessary to adapt shorter versions of existing scales. Scale adaptations also included broadly defining citizen science and using a single item to measure participation, which left room for misinterpretation of citizen science participation. We tried to account for this in analysis by making distinctions between multi-project and single-project participants, with the assumption that multi-project participants were more familiar with the citizen science activities and more likely to have participated in formal citizen science projects.

Additionally, our research attempted to conceptualize and measure a complex, dynamic representation of the public’s relationship with science at a single point in time. Longitudinal studies that monitor changes in science beliefs and connections over time could reveal insightful patterns, but more work is needed to establish consistent survey methods and instruments for long-term measurement of these latent constructs. Long-running assessments like the National Science Board’s Science and Engineering Indicators survey are one method for doing this; our study suggests that incorporating affective dimensions of people’s relationship with science (e.g., belonging, identity) could be valuable dimensions to these measures. Our study did not find differences in science beliefs or connections between urban and rural respondents, likely due to inadequate statistical power because our sample was 90% urban. But evidence of urban-rural divides related to political or religious beliefs and science in America may be growing [117,118] and future research might collect additional demographic data to examine that relationship in more detail.

More longitudinal, experimental, and qualitative studies are needed to continue exploring the capacity for citizen science participation to influence science beliefs and connections across diverse populations. Without directly testing participation as an intervention, it is difficult to confirm a causal relationship between citizen science participation and positive outcomes, especially when many participants self-select to volunteer based on inherent affinities for science [115]. Future work could strive to identify citizen science project characteristics or experiences that bolster participants’ relationship to science and explore how they could better engage audiences who are new to science.

## Conclusions

Overall, despite concerns about the politicization of science exacerbated by the COVID-19 pandemic, Americans’ relationship with science generally appeared to remain positive. A majority of Americans are interested in science and believe that science has value to society, and these numbers have not fluctuated drastically over the past 50 years. However, a significantly smaller portion of the population expresses a sense of belonging in science or science identity, and less than half of the U.S. population viewed science as credible at the time of our study. Implications of these weaker relationships could include a decline in public support for scientific research or funding, perceptions of scientists’ credibility, and cooperation with scientific recommendations during times of crisis [119] – this is particularly concerning when coupled with the politicization of science and message frames that activate political identities as either “pro-” or “anti-” science [120]. Disparities in beliefs and connections to science among different demographic groups is also a concern. Individuals without a college degree and Black Americans are significantly less likely to believe science has value to society, underscoring the need to increase access and inclusion in the sciences. To ensure the most vulnerable segments of society see themselves as beneficiaries of the social, environmental, and technological progress of science, we must create a more diverse and equitable scientific community.

Citizen science is one tool that could help strengthen or reinforce Americans’ positive relationship with science. Individuals who participate in citizen science projects express more positive beliefs about science and stronger connections to science, though causality in this relationship remains unknown. Research investigating factors that cultivate public trust in science will be key moving forward. Citizen science interventions offer a promising option, but to generate widespread social impact and achieve its transformative potential, citizen science efforts must expand beyond typical audiences to engage communities historically excluded and alienated from science.

## Supporting information

S1 Table Multi-factor Structure of Items Measuring Science Beliefs Among U.S. respondents (n = 1335). Exploratory factor analysis conducted with Principal Axis Factoring and oblique (Promax) rotation; KMO = 0.674. Highest factor loadings for pattern matrix and structure matrix are in bold. Two factors with eigenvalues > 1.0 (Factor 1 = 2.1, 34.9% of cumulate variance explained, Factor 2 = 2.0, 33.6%, r = 0.016). a Items rated on scale: −2 = Strongly disagree, −1 = Disagree, 0=Neither, 1 = Agree, 2 = Strongly agree. Means are unweighted.
(PDF)

S2 Table Multi-factor Structure of Items Measuring Science Connections Among U.S. Respondents (n = 1854). Exploratory factor analysis conducted with Principal Axis Factoring and oblique (Promax) rotation; KMO = 0.880. Highest factor loadings for pattern matrix and structure matrix are in bold. Two factors with eigenvalues > 1.0 (Factor 1 = 4.0, 50.5% of cumulate variance explained, Factor 2 = 1.4, 17.3%, r = 0.250). Two reverse-coded items included on the original scale were removed prior to factor analysis (I am not interested in science, I do not enjoy doing science activities). a Items rated on scale: −2 = Strongly disagree, −1 = Disagree, 0=Neither, 1 = Agree, 2 = Strongly agree. Means are unweighted.
(PDF)

S3 Table Parameter Estimates for Demographic Predictors of Science Beliefs Among U.S. respondents (n = 1297). Parameter estimates (B), standard errors (SE), and standardized parameter estimates (β) in science beliefs linear regression models predicting the two components of science beliefs – (a) perceived value of science and (b) perceived credibility of science. *, **, *** denote statistical significance of parameter estimate at α = 0.05, 0.01, and 0.001, respectively. a Perceived Value of Science Model Details: M = 0.61 (SD = 0.83); 3 items rated on scale from −2 = Strongly disagree to +2 = Strongly agree; Model fit statistics: F(14,1282) = 10.39, p < 0.001, Adjusted R^2^ = 0.092. b Perceived Credibility of Science Model Details: M = 0.10 (SD = 0.94); 3 items rated on scale from −2 = Strongly disagree to +2 = Strongly agree; Model fit statistics: F(14,1282) = 2.86, p < 0.001, Adjusted R^2^ = 0.020.
(PDF)

S4 Table Parameter Estimates for Demographic Predictors of Science Connections Among U.S. respondents (n = 1807). Parameter estimates (B), standard errors (SE), and standardized parameter estimates (β) in the linear regression models predicting two components of science connections – (a) science interest and self-efficacy and (b) science belonging. *, **, *** denote statistical significance of parameter estimate at α = 0.05, 0.01, and 0.001, respectively. ^a^Science Interest and Self-Efficacy Model Details: M = 0.38 (SD = 0.88); 6 items rated on scale from −2 = Strongly disagree to +2 = Strongly agree; Model fit statistics: F(14,1792) = 18.59, p < 0.001, Adjusted R^2^ = 0.120. ^b^Science Belonging Model Details: M = 0.12 (SD = 1.04); 2 items rated on scale from −2 = Strongly disagree to +2 = Strongly agree; Model fit statistics: F(14,1792) = 2.65, p < 0.001, Adjusted R^2^ = 0.013.
(PDF)

S5 Table Parameter Estimates for Demographic Predictors of Science Identity Among U.S. Respondents (n = 1807). Parameter estimates (B), standard errors (SE), and odds ratios (OR) for variables in the logistic regression model predicting self-reported science identity. *, **, *** denote statistically significant Odds Ratio (OR) at α = 0.05, 0.01, and 0.001, respectively. Unweighted percentage of participants responding “Yes” to “Do you identify as a scientist?”: 17.4%. Model Fit Statistics: n = 1,807; Cragg-Uhler (Nagelkerke) *R*^*2*^: 0.345, Classification Accuracy: 82.7%, χ^2^(14) = 421.5, *p* < 0.001.
(PDF)

S1 Survey Instrument Full Questionnaire Administered Through Qualtrics XM to U.S. Residents in 2021. The survey for our study was part of a broader national survey effort to collect information about public perceptions and engagement with citizen science. As the scope of our study focuses on beliefs and connections the public hold towards science – not citizen science, specifically – and how citizen science participation is linked, our data is comprised from responses to questions on pages 5–11, 22–23, and 29–38 this survey.
(PDF)
