# Supplementary material for: Americans’ multidimensional relationship with science: Is citizen science a path forward?
Source: PLoS One. 2026 Sep 11;21(9):e0357476. doi: 10.1371/journal.pone.0357476 (PMC13567779; doi:10.1371/journal.pone.0357476)
Supplement: Supplementary file 1 [file pone.0357476.s001.pdf]

Index/Items[a]	Mean	SD	Pattern Matrix		Structure Matrix	
			Fac. 1	Fac. 2	Fac. 1	Fac. 2
**1. Value of Science** (3 items; Cronbach's α = .750)	**.60**	**.84**				
Science and technology make life healthier, easier, and more comfortable	.68	1.01	**.767**	.051	**.768**	.063
Even if it brings no immediate benefits, scientific research is necessary and should be supported by the government	.67	1.04	**.752**	.003	**.752**	.015
Overall, the benefits of scientific research have outweighed any harmful effects	.45	1.01	**.609**	-.077	**.608**	-.067
**2. Credibility of Science** (3 items; Cronbach's α = .780)	**.11**	**.94**				
Our society places too much emphasis on science (reverse coded)	.10	1.12	.003	**.752**	.039	**.753**
People trust scientists a lot more than they should (reverse coded)	.10	1.11	-.113	**.685**	-.102	**.684**
Sometimes I think we put too much faith in science (reverse coded)	.09	1.15	-.077	**.609**	.072	**.777**
