# Supplementary material for: Americans’ multidimensional relationship with science: Is citizen science a path forward?
Source: PLoS One. 2026 Sep 11;21(9):e0357476. doi: 10.1371/journal.pone.0357476 (PMC13567779; doi:10.1371/journal.pone.0357476)
Supplement: Supplementary file 2 [file pone.0357476.s002.pdf]

Index/Items[a]	Mean	SD	Pattern Matrix		Structure Matrix	
			Fac. 1	Fac. 2	Fac. 1	Fac. 2
**1. Science Interest & Efficacy** (6 items; Cronbach's α = .891)	**.38**	**.88**				
I think I'm good at following scientific research protocols	.32	1.09	**.779**	-.025	**.771**	.222
I enjoy participating in scientific projects and studies	.38	1.10	**.765**	-.017	**.760**	.225
I think of myself as someone who can contribute to science	.32	1.09	**.764**	.023	**.771**	.265
I like talking about science topics with others	.32	1.11	**.750**	.001	**.751**	.239
I feel confident about my ability to contribute to scientific studies	.27	1.10	**.749**	.020	**.755**	.257
I like learning about science	.63	1.08	**.747**	-.002	**.746**	.234
**2. Science Belonging** (2 items; Cronbach's α = .692)	**.12**	**1.04**				
I do not see myself as part of the larger scientific community (reverse coded)	.05	1.18	-.057	**.745**	.179	**.727**
I feel like I do not belong in science (reverse coded)	.20	1.20	.061	**.720**	.289	**.739**
