# Supplementary material for: Americans’ multidimensional relationship with science: Is citizen science a path forward?
Source: PLoS One. 2026 Sep 11;21(9):e0357476. doi: 10.1371/journal.pone.0357476 (PMC13567779; doi:10.1371/journal.pone.0357476)
Supplement: Supplementary file 3 [file pone.0357476.s003.pdf]

		Perceived Value of Science[a]			Perceived Credibility of Science[b]		
**Variables**	**Mean**	**B**	**SE**	**β**	**B**	**SE**	**β**
Gender (man)	0.33	0.166	0.047	0.095***	0.014	0.056	0.007
Race (ref. = White)	0.14						
Hispanic	0.24	0.015	0.075	0.008	-0.064	0.088	-0.029
Black	0.21	-0.302	0.076	-0.149***	-0.008	0.090	-0.003
Asian	0.26	0.071	0.074	0.038	-0.081	0.087	-0.038
American Indian	0.07	-0.139	0.103	-0.041	0.009	0.122	0.002
Multiracial/Other	0.08	-0.164	0.096	-0.054	0.267	0.114	0.077*
Age (ref. = 18-34 years)	0.49						
35-49 years	0.32	0.123	0.051	0.069*	-0.106	0.060	-0.052
50+ years	0.19	0.296	0.062	0.141***	-0.062	0.073	-0.026
Education (ref. = high school)	0.45						
College degree	0.41	0.177	0.050	0.105***	0.076	0.059	0.039
Graduate degree	0.14	0.195	0.072	0.082**	0.057	0.085	0.021
STEM profession (yes)	0.37	-0.064	0.049	-0.037	-0.156	0.058	-0.080**
Current location (urban)	0.88	0.102	0.070	0.040	0.141	0.083	0.048
Citizen science participation (ref. = none)	0.66						
Single project participant	0.21	0.129	0.057	0.064*	-0.162	0.067	-0.070*
Multi-project participant	0.13	0.327	0.070	0.132***	-0.225	0.083	-0.080**
