# Supplementary material for: Americans’ multidimensional relationship with science: Is citizen science a path forward?
Source: PLoS One. 2026 Sep 11;21(9):e0357476. doi: 10.1371/journal.pone.0357476 (PMC13567779; doi:10.1371/journal.pone.0357476)
Supplement: Supplementary file 4 [file pone.0357476.s004.pdf]

		(a) Science Interest & Self-efficacy[a]			(b) Science Belonging[b]		
**Variables**	**Mean**	**B**	**SE**	**β**	**B**	**SE**	**β**
Gender (man)	0.36	0.130	0.041	0.071**	0.069	0.052	0.032
Race (ref. = White)	0.26						
Hispanic	0.20	0.106	0.059	0.048	0.098	0.075	0.038
Black	0.20	-0.010	0.059	-0.005	0.097	0.075	0.037
Asian	0.21	0.125	0.059	0.058*	0.162	0.074	0.063*
American Indian	0.05	0.084	0.097	0.020	0.125	0.122	0.026
Multiracial/Other	0.08	0.054	0.081	0.016	0.207	0.102	0.052*
Age (ref. = 18-34 years)	0.49						
35-49 years	0.31	0.181	0.046	0.095***	0.078	0.057	0.035
50+ years	0.20	0.037	0.054	0.017	-0.005	0.068	-0.002
Education (ref. = high school)	0.45						
College degree	0.40	0.069	0.044	0.038	0.098	0.055	0.046
Graduate degree	0.15	0.185	0.062	0.075**	0.042	0.056	0.013
STEM profession (yes)	0.39	0.116	0.044	0.064**	0.027	0.056	0.013
Current location (urban)	0.87	-0.009	0.060	-0.003	0.127	0.075	0.041
Citizen science participation (ref. = none)	0.65						
Single project participant	0.22	0.356	0.050	0.168***	-0.004	0.063	-0.002
Multi-project participant	0.13	0.669	0.062	0.256***	0.274	0.078	0.089***
