# Supplementary material for: Americans’ multidimensional relationship with science: Is citizen science a path forward?
Source: PLoS One. 2026 Sep 11;21(9):e0357476. doi: 10.1371/journal.pone.0357476 (PMC13567779; doi:10.1371/journal.pone.0357476)
Supplement: Supplementary file 5 [file pone.0357476.s005.pdf]

		Science Identity		
**Variables**	**Mean**	**B**	**SE**	**OR**
Gender (man)	0.36	0.129	0.146	1.14
Race (ref. = White)	0.26			
Hispanic	0.20	0.456	0.223	1.59*
Black	0.20	0.312	0.218	1.36
Asian	0.21	-0.079	0.229	0.92
American Indian	0.05	0.003	0.418	1.00
Multiracial/Other	0.08	0.098	0.294	1.10
Age (ref. = 18-34 years)	0.49			
35-49 years	0.31	-0.420	0.168	0.66*
50+ years	0.20	-0.536	0.235	0.59*
Education (ref. = high school)	0.45			
College degree	0.40	-0.100	0.168	0.91
Graduate degree	0.15	0.548	0.200	1.73**
STEM profession (yes)	0.39	2.324	0.178	10.22***
Current location (urban)	0.87	0.035	0.233	1.04
Citizen science participation (ref. = none)	0.65			
Single project participant	0.22	0.773	0.167	2.17***
Multi-project participant	0.13	0.888	0.193	2.43***
