# Supplementary material for: Americans’ multidimensional relationship with science: Is citizen science a path forward?
Source: PLoS One. 2026 Sep 11;21(9):e0357476. doi: 10.1371/journal.pone.0357476 (PMC13567779; doi:10.1371/journal.pone.0357476)
Supplement: Supplementary file 6 [file pone.0357476.s006.pdf]

Public Perspectives on "Citizen Science"

Note: Section 3 (start page 29) contains items for measuring science beliefs and science connections.

Survey Flow

EmbeddedData
opp = Qual5859-0812-newsurveyonpublicperspectivesoncitizenscience
RISNValue will be set from Panel or URL.
ridValue will be set from Panel or URL.
VValue will be set from Panel or URL.
Qpmid = 73551282
Q_TotalDurationValue will be set from Panel or URL.
cintidValue will be set from Panel or URL.
ProjectToken = 96e1c5c0-4ea9-95c5-ede7-28fa3fc3fe1b
gidValue will be set from Panel or URL.
snameValue will be set from Panel or URL.
vigaValue will be set from Panel or URL.
CMRIDValue will be set from Panel or URL.

Standard: Introduction (2 Questions)

Branch: New Branch
If
If Quota Overall N 1300 Has Been Met

EmbeddedData
gc = 3
term = OVQ

EndSurvey: Advanced

Branch: New Branch
If
If I have read the information above and I agree to participate in this survey. No Is Selected

EmbeddedData
gc = 2
term = consent

EndSurvey: Advanced

Standard: Block 5 (3 Questions)

Branch: New Branch
If
If What is your country of residence? (Select ONE response.) United States Is Selected
AndIf
If Are you a citizen of the United States? (Select ONE response.) Yes, born in the United States or U.S. territory (Puerto Rico, Guam, etc.) Is Selected

Or Are you a citizen of the United States? (Select ONE response.) Yes, born abroad of U.S. citizen parent(s) Is Selected
Or Are you a citizen of the United States? (Select ONE response.) Yes, U.S. citizen by naturalization Is Selected

EmbeddedData
quota = US_cit

Block: Participant Information (8 Questions)

Branch: New Branch
If
If What is your country of residence? (Select ONE response.) United States Is Selected
AndIf
If Are you a citizen of the United States? (Select ONE response.) No, not a U.S. citizen Is Selected
Or Are you a citizen of the United States? (Select ONE response.) Other (please specify): Is Selected

EmbeddedData
quota = US_non_cit

Branch: New Branch
If
If What is your country of residence? (Select ONE response.) United States Is Not Selected

EmbeddedData
quota = US_non_cit

Standard: Describing Public Participation in Science (12 Questions)
Standard: Constraints to Participating (5 Questions)
Standard: Connection to Science (6 Questions)

EmbeddedData
Email = ${m://Email1}

Branch: New Branch
If
If Q_TotalDuration Is Equal to 90

EmbeddedData
gc = 4
term = Speeder

EndSurvey: Advanced

Branch: New Branch
    If
        If Quota Overall N 1300 Has Been Met

EmbeddedData
    gc = 3
    term = OVQ

EndSurvey: Advanced

EmbeddedData
    gc = 1
    LS = $e{(${e://Field/RISN}%3402)*3}
    ProjectToken = c79387fc-485e-4ccb-bcb4-a1f8c6f48283
    TolunaEnc = $e{ ((e://Field/gid + 3) % 4001) * 17 }

EndSurvey: Advanced

Page Break

Start of Block: Introduction

Intro **Public Participation in Science: What's Your Perspective?** Around the world, **more and more people are participating in science, whether they are scientists or not.** Here are some of the activities where public involvement in science is growing: Identifying issues for study Designing experiments Collecting information or data Conducting analyses Interpreting study results Communicating those results to others **Our research team would like to learn what people around the world think about different phrases that describe this process of people, including those without scientific credentials, contributing to science.** Your participation in this study is voluntary, and you may elect to skip any question. Your responses are anonymous, and they will be combined with all of the other responses so that no one person will ever be identifiable. Collective responses will be shared with people interested in science and the public, including professors, project leaders, and community organizers. Our team will also seek to summarize the results in one or more publications. Anyone 18 years of age or older can participate in this survey. The study is being conducted by a research team at North Carolina State University. If you have any questions or would like to learn more about the study, please contact Dr. Caren Cooper by email at cbcoope3@ncsu.edu. If you have any questions regarding your rights as a subject in this study, you may contact the Institutional Review Board (IRB) for Human Participants Administrator at NC State University at 1-919-515-4514. **We sincerely hope you will take 10-15 minutes to answer our questions.** Thank you for your help!

Consent I have read the information above and I agree to participate in this survey.

- ◯ Yes, take me to the survey. (1)
- ◯ No (2)

Skip To: End of Block If Consent = 2

Page Break

End of Block: Introduction

Start of Block: Block 5

Display this question:

If If False

Q4.7 **What is your country of residence?** *(Select ONE response.)*

○ United Kingdom (1)

○ United States (2)

○ Other (please specify): (3) ____

Skip To: End of Block If Q4.7 != 2

Page Break

Display this question:

If If False

Q4.7b **Are you a British citizen?** *(Select ONE response.)*

◯ Yes (1)

◯ No (2)

Display this question:

If If False

Q4.7c **What is your country of birth?** *(Select ONE response.)*

◯ England (1)

◯ Northern Ireland (2)

◯ Republic of Ireland (3)

◯ Scotland (4)

◯ Wales (5)

◯ Elsewhere (write in country): (6) ____

End of Block: Block 5

Start of Block: Participant Information

Sec4 **First, please tell us a little about yourself.**

Q4.1 **With what gender do you identify?** (Select ALL that apply.)

- ▢ Man (1)
- ▢ Woman (2)
- ▢ Non-binary (3)
- ▢ Other/not listed (specify): (4) ____

Q4.2 **Which of the following best describes your racial/ethnic background?** (Select *ALL that apply.)*

- ☐ White (1)
- ☐ Hispanic/Latino (2)
- ☐ Black or African American (3)
- ☐ Asian (4)
- ☐ American Indian or Alaska Native (5)
- ☐ Middle Eastern or North African (6)
- ☐ Native Hawaiian or Other Pacific Islander (7)

Display this choice:

If If False

- ☐ Mixed Race (9)
- ☐ Other (specify): (8) ____

Q4.3 Is English your primary language?

- ○ Yes (1)
- ○ No (please list other language(s) here): (2) ____

Q4.4 **In what year were you born?** (Write YEAR in space provided.)

- ○ Year of Birth: (1) ____

Q4.7a **Are you a citizen of the United States?** *(Select ONE response.)*

- ○ Yes, born in the United States or U.S. territory (Puerto Rico, Guam, etc.) (1)
- ○ Yes, born abroad of U.S. citizen parent(s) (2)
- ○ Yes, U.S. citizen by naturalization (3)
- ○ No, not a U.S. citizen (4)
- ○ Other (please specify): (7)

____

Q4.5 **What is the highest level of education that you have completed?** *(Please select ONE response that best describes your highest level of education.)*

- ○ Some high school or secondary school (1)
- ○ High school or secondary school diploma or GED (2)
- ○ Associate's or Bachelor's degree (BA, BS, etc.) (3)
- ○ Graduate or professional degree (MS, MBA, MD, JD, PhD, etc.) (4)

Q4.6 **Which of the following categories BEST describes your primary occupation or professional field?** *(Mark ONE and specify as needed. If you are a student, a home-maker, or*

someone who is retired or unemployed, please indicate the option that best describes the field of your professional training.)

◯ STEM field or profession (science, technology, engineering, or math) (1)

◯ Other NON-STEM field or profession (not science, technology, engineering, or math) (2)

Page Break

End of Block: Participant Information

Start of Block: Describing Public Participation in Science

Sec1 **Below you will see several phrases that might be used to describe the process of people, including those without scientific credentials, contributing to science.** These contributions might include activities such as: Identifying issues for study Designing experiments Collecting information or data Conducting analyses Interpreting study results Communicating those results to others

Q1.1 Which of the following phrases have you heard before today? (Check ALL that apply.)

- ▢ Citizen science (1)
- ▢ Civic science (6)
- ▢ Community science (2)
- ▢ Crowdsourcing (7)
- ▢ Participatory research (5)
- ▢ Public science (4)
- ▢ Volunteer science (3)
- ▢ I have NOT heard any of these phrases (8)
- ▢ I have heard OTHER phrases that describe these activities (please specify): (9) ____

Page Break

Q1.2 **In your opinion, how well does each phrase below describe the process of people, including those without scientific credentials, contributing to science?** *(Select ONE response for each item.)*

	Very poor description (-2)	Poor description (-1)	Fair description (0)	Good description (1)	Excellent description (2)
Citizen science (1)	○	○	○	○	○
Civic science (6)	○	○	○	○	○
Community science (2)	○	○	○	○	○
Crowdsourcing (7)	○	○	○	○	○
Participatory research (5)	○	○	○	○	○
Public science (4)	○	○	○	○	○
Volunteer science (3)	○	○	○	○	○

Q1.2a Which phrase below BEST describes the process of people, including those without scientific credentials, contributing to scientific research activities? Which phrase below offers the WORST description of these activities? *(Select one response for the "BEST description" AND one response for the "WORST description" column.)*

BEST description (1)		WORST description (0)

○	⊗ Citizen science (6)	○
○	⊗ Civic science (12)	○
○	⊗ Community science (7)	○
○	⊗ Crowdsourcing (11)	○
○	⊗ Participatory research (8)	○
○	⊗ Public science (9)	○
○	⊗ Volunteer science (10)	○

Page Break

Display this question:

If If False

Q1.3 **How comfortable do you feel using each of the phrases below?** *(Select ONE response for each item.)*

	Very uncomfortable (-2)	Uncomfortable (-1)	Neither comfortable or uncomfortable (0)	Comfortable (1)	Very comfortable (2)
Citizen science (1)	○	○	○	○	○
Civic science (7)	○	○	○	○	○
Community science (2)	○	○	○	○	○
Crowdsourcing (6)	○	○	○	○	○
Participatory research (5)	○	○	○	○	○
Public science (4)	○	○	○	○	○
Volunteer science (3)	○	○	○	○	○

Q1.4 **How included do you feel in each of the phrases below?** *(Select ONE response for each item.)*

	Very excluded (-2)	Excluded (-1)	Neutral (0)	Included (1)	Very included (2)
Citizen science (1)	○	○	○	○	○
Civic science (7)	○	○	○	○	○
Community science (2)	○	○	○	○	○
Crowdsourcing (6)	○	○	○	○	○
Participatory research (5)	○	○	○	○	○
Public science (4)	○	○	○	○	○
Volunteer science (3)	○	○	○	○	○

Q1.4a Which phrase below describes an activity that sounds the MOST inclusive? Which phrase describes an activity that sounds the LEAST inclusive? *(Select one response for the "most inclusive" AND one response for the "least inclusive" column.)*

MOST inclusive (1)		LEAST inclusive (0)

○	⊗ Citizen science (6)	○
○	⊗ Civic science (12)	○
○	⊗ Community science (7)	○
○	⊗ Crowdsourcing (11)	○
○	⊗ Participatory research (8)	○
○	⊗ Public science (9)	○
○	⊗ Volunteer science (10)	○

Page Break

Q1.5 **How likely are you to engage in activities described by each of the following phrases?** *(Select ONE response for each item.)*

	Very unlikely to engage (-2)	Unlikely to engage (-1)	Not sure (0)	Likely to engage (1)	Very likely to engage (2)
Citizen science (1)	○	○	○	○	○
Community science (2)	○	○	○	○	○
Crowdsourcing (7)	○	○	○	○	○
Civic science (8)	○	○	○	○	○
Participatory research (5)	○	○	○	○	○
Public science (4)	○	○	○	○	○
Volunteer science (3)	○	○	○	○	○

Q1.5a Which phrase below describes an activity you would be MOST likely to engage in? Which phrase describes an activity you would be LEAST likely to engage in?
(Select one response for the "most likely to engage in" AND one response for the "least likely to engage in" column.)

MOST likely to engage (1)		LEAST likely to engage (2)

○	⊗ Citizen science (6)	○
○	⊗ Civic science (12)	○
○	⊗ Community science (7)	○
○	⊗ Crowdsourcing (11)	○
○	⊗ Participatory research (8)	○
○	⊗ Public science (9)	○
○	⊗ Volunteer science (10)	○

Page Break

Q1.6 **Overall, in your opinion, which ONE of the following phrases do you think should be used to describe the process of people, including those without scientific credentials, contributing to science?** (Select ONE response.)

- ◯ Citizen science (1)
- ◯ Civic science (6)
- ◯ Community science (2)
- ◯ Crowdsourcing (7)
- ◯ Participatory research (5)
- ◯ Public science (4)
- ◯ Volunteer science (3)
- ◯ Other (please specify): (8) ____

Display this question:

If If False

Q1.6a **Please explain your reasoning.** *(Write answer in the space provided.)*

____

____

____

____

____

Q1.6b **Do you have concerns about any of the phrases listed above? Why?**
(Write answer in the space provided.)

____

Page Break

End of Block: Describing Public Participation in Science

Start of Block: Constraints to Participating

Display this question:

If hide = hide

X→

Q2.1OLD
When thinking about the relationship between the practice of science and the general public (including both scientists and those without scientific credentials), which of the following best describes you? *(Please select ONE response that best describes your involvement.)*

◯ I have engaged as a practitioner (i.e., creator or manager of a science project or program) (1)

◯ I have engaged as a participant (i.e., someone contributing to a science project or program) (2)

◯ I have engaged as BOTH a practitioner and a participant (3)

◯ I have NOT engaged in these types of activities (4)

◯ I am NOT SURE if I have engaged in these types of activities (5)

X→

Q2.1
Select ONE response that best describes your involvement in the activities described earlier in the survey (citizen science, civic science, community science, crowdsourcing,

participatory research, public science, and/or volunteer science)?
(Please select ONE response that best describes your involvement.)

○ I have NOT engaged in these types of activities (1)

○ I am NOT SURE if I have engaged in these types of activities (2)

○ I have engaged as a leader or coordinator of participants in these activities (3)

○ I have engaged as a participant in these activities (4)

○ I have both led/coordinated AND participated in these activities (5)

Display this question:

If Q2.1 = 3

Or Q2.1 = 4

Or Q2.1 = 5

X→

Q2.1a
Which of the following types of scientific research projects have you been involved in?
(Select ALL that apply. If you aren't sure where the projects you've done might fit, just take your best guess.)

▢ Behavioral & social science projects (1)

▢ Environmental & life science projects (2)

▢ Engineering & earth/physical science projects (3)

▢ Health & medical science projects (4)

▢ Other types of science projects (please specify): (5)
____

Page Break

Q2.2a
To what extent are each of the following items barriers or reasons that you do not contribute (or do not contribute more often) to science?
(Select ONE response for each item.)

	Is this a barrier or reason you do not contribute to science?			
	Not a barrier (0)	Minor barrier (1)	Moderate barrier (2)	Major barrier (3)

I lack free time to participate (1)	○	○	○	○
I am not interested in doing science activities (2)	○	○	○	○
Participating in science activities is not fun for me (19)	○	○	○	○
I don't have the skills or abilities to do science activities well (4)	○	○	○	○
Science activities don't accommodate my disability (20)	○	○	○	○
Science activities aren't available in my primary language (22)	○	○	○	○
I am not aware of opportunities to participate (5)	○	○	○	○
I do not have access to these opportunities (9)	○	○	○	○
I was never invited to engage in science activities (24)	○	○	○	○
The cost of science activities is too high (3)	○	○	○	○
I lack the training needed to do science activities (10)	○	○	○	○

I lack the technological resources needed to do science activities (17)	○	○	○	○
I don't have people to participate with me (25)	○	○	○	○
I lack support from family and/or peers (26)	○	○	○	○
Science activities don't align with my values (27)	○	○	○	○
Science activities aren't designed for people like me (28)	○	○	○	○
I don't feel welcome in science activities (29)	○	○	○	○
I don't feel comfortable due to a lack of diversity in science activities (30)	○	○	○	○
I fear negative consequences from my participation in science activities (31)	○	○	○	○

Science activities won't help me address problems I care about (32)	○	○	○	○
My participation won't make a difference (33)	○	○	○	○

Q2.2b If you have experienced barriers, or have reasons you don't contribute (or do not contribute more often) to scientific research activities, please briefly explain the most serious or significant ones. (Write answer in the space provided.)

____

____

____

____

____

Page Break

End of Block: Constraints to Participating

Start of Block: Connection to Science

Sec3 **Next, please tell us a little more about your connection to science...**

Q3.1 To what extent do you disagree or agree with the following statements?
(Select ONE response for each item.)

	Strongly disagree (-2)	Disagree (-1)	Neither agree nor disagree (0)	Agree (1)	Strongly agree (2)
I like learning about science (1)	○	○	○	○	○
I am not interested in science (2)	○	○	○	○	○
I enjoy participating in scientific projects and studies (3)	○	○	○	○	○
I do not enjoy doing science activities (10)	○	○	○	○	○
I like talking about science topics with others (6)	○	○	○	○	○
I feel confident about my ability to contribute to scientific studies (4)	○	○	○	○	○
I think I'm good at following scientific research protocols (5)	○	○	○	○	○
I feel like I do not belong in science (7)	○	○	○	○	○

I think of myself as someone who can contribute to science (9)	○	○	○	○	○
I do not see myself as part of a larger scientific community (8)	○	○	○	○	○

Page Break

Q3.2 **Do you identify as a scientist?** *(Select ONE response.)*

- Yes (1)
- No (6)
- Not sure (7)

Display this question:

If Q3.2 = 1

Or Q3.2 = 7

Q3.2a **To what extent do you identify as a scientist?** *(Select ONE response.)*

- Slightly (1)
- Moderately (2)
- Strongly (3)
- Very strongly (4)

Page Break

Q3.3 **To what extent do you agree with the following statements about science?**
(Select ONE response for each item.)

	Strongly disagree (-2)	Disagree (-1)	Neither agree nor disagree (0)	Agree (1)	Strongly agree (2)
People trust scientists a lot more than they should (1)	○	○	○	○	○
Science and technology make life healthier, easier and more comfortable (2)	○	○	○	○	○
Sometimes I think we put too much faith in science (3)	○	○	○	○	○
Overall, the benefits of scientific research have outweighed any harmful effects (4)	○	○	○	○	○
Our society places too much emphasis on science (5)	○	○	○	○	○

Even if it brings no immediate benefits, scientific research is necessary and should be supported by the government (6)	○	○	○	○	○

Page Break

End **Thanks for taking the time to complete this survey. Please click the forward arrow to submit your responses.**

End of Block: Connection to Science
