# Supplementary material for: Americans’ multidimensional relationship with science: Is citizen science a path forward?
Source: PLoS One. 2026 Sep 11;21(9):e0357476. doi: 10.1371/journal.pone.0357476 (PMC13567779; doi:10.1371/journal.pone.0357476)
Supplement: Supplementary file 7 [file pone.0357476.s008.docx]

**Manuscript PONE-D-26-06030**

**Response to Reviewers**

Dear Floris Vermeulen,

Thank you for the opportunity to submit a revised draft of our manuscript titled “Americans’ multidimensional relationship with science: Is citizen science a path forward?” for publication in *PLOS One.* We appreciate the time and effort that went into reviewing our manuscript and the thoughtful comments provided by reviewers to improve our paper. We have addressed all suggestions made by the reviewers and provide a point-by-point response below. All page numbers refer to the revised manuscript file with tracked changes. Additionally, we have addressed the formatting and data availability issues brought to our attention and summarized those changes at the end of this letter.

**Reviewers’ Comments to the Authors:**

**Reviewer 1**

The authors clearly state why their research is relevant and provide detailed information on the methods that they have employed. In addition, the manuscript is well-structured.

**Author response:** Thank you for this feedback!

1. Comment regarding expansion on the definition of citizen science as a concept.

**Author response:** This was an excellent point that did relate to a point in our analysis of open-ended response data where some respondents interpreted their survey participation as a citizen science activity. As you suggested, we have added additional context to our definition of citizen science, specifically including criteria and additional citations for what activities constitute citizen science, including why survey research is not citizen science (lines 196 – 203).

The new Intro text says:

“Considering the broad engagement described above, we define *citizen science participation* as a process where members of public, including non-scientists, participate in one or more of the following scientific activities: identification of issues for study, experimental design, collection of data, data analyses, interpretation of study results, and communication of those results to others. Though the degree of public participation in a project may vary, an emphasis on public interest and impact beyond their participation as research subjects is a defining principle of citizen science [71]. Thus, participation in survey and clinical trial research is often excluded as citizen science practice [78].”

2. Clarification for what demographic group(s) were compared in the text supporting Figure 3: “Black Americans were significantly less likely than others to report science has value to society”.

**Author response:** Thank you for pointing this out; here we were comparing results between Black Americans to the reference category of White Americans. We have changed the text to read “Black Americans were significantly *less* likely to report that science has value to society (β = -0.149, p < 0.001) compared to White Americans” (line 347).

3. Possibly adding effect sizes to the results section to help interpret significant findings.

**Author response:** This was helpful to point out and did help us catch a typo in the reporting of these results. We updated the regression results in text to clarify that we were reporting relative effect sizes (standardized β) and updated the captions of fig 3 and fig 4 to clarify that the graphs represent *un*standardized B coefficients in order to calculate 95% confidence intervals (lines 343 – 368). Researchers often use standardized beta to highlight relative effect sizes in regression models (Nieminen, 2022), so this clarification should address the reviewer’s concern and facilitate interpretation of results, helping readers understand which predictor variables matter the most.

Nieminen, P. (2022). Application of standardized regression coefficient in meta-analysis. *BioMedInformatics*, *2*(3), 434-458.

**Reviewer 2**

The theory, methods and results are developed well. Only a few revisions are needed to make the article clearer for the readers.

**Author response:** Thank you for your feedback!

1. The definition of science, its mechanism explanation, and alignment with the questionnaire design.

**Author response:** After reviewing your feedback, we do agree that the multiple definitions of science are somewhat misaligned with how they are interpreted and operationalized, particularly between how we (the researchers) define science, how we believe the public might define science, and how we operationalize measures related to science in this work. We expanded our definition of science to address concept versus practice in our introduction (lines 87 – 96) and in the methods section describing the questionnaire design (239 – 243). In this later section, we also address how in our study the scope of interest was more in how individuals perceived their relationship with science, subjectively, rather than how they might define science. Ultimately, we wanted to people to respond to questions with their own view/definition of science in mind, not a formal definition prescribed by the researchers.

Specifically, we added this explanation in Methods:

“Because our survey asked people to reflect on their personal relationship with science, we did not include a formal definition of “science” that could potentially limit how respondents interpreted and answered survey items about their science beliefs and connections. This allowed respondents to describe personal actions, feelings, and beliefs about science according to their own terms.”

2. Definition and mention of the Western origin of science.

**Author response:** This is a good point. We felt it was important to acknowledge the broader cultural paradigm that influences how people in the US might conceptualize science in concept and practice, as how they interpret science may influence their relationship with it. But (as pointed out by the reviewer) it’s important to acknowledge that people with non-western origins may have differing beliefs and connections with science compared to a majority of Americans, which we added in lines 93 – 96. A deeper investigation of these different cultural perceptions of science is beyond the scope of this study, however.

Lines 93 – 96 now read:

“How people personally understand and define ‘science’ is also likely to vary across a diverse public encompassing people of varying demographic backgrounds (including non-Western origins), experiences, and access to scientific information.”

3. Definition and subjectivity of STEM

**Author response:** We agree that interpretations of STEM and what constitutes STEM may vary across respondents, though we tried to provide some clarity in our questionnaire by adding examples of STEM disciplines in parentheses (science, technology, engineering, or math). We added a note on this in the limitations of our questionnaire design and discussed how that might also relate to the reviewers next comment about subjective science identity (lines 560 – 562).

4. Operationalization of science identity measure

**Author response:** This is an important clarification, and we have updated main text (line 253) and limitations (lines 560-562) to clarify that we are measuring a subjective sense of science identity that is not indicative of respondents’ self-described STEM occupation (and vice versa).

Lines 560-562 now say:

“Respondents' science identity and professional STEM affiliations are also subjective and more complex than our single-item measures could capture. As our study showed, for instance, STEM affiliation does not necessarily indicate science identity.”

5. Including discussion on the difference between idealized science and science as a practice in the results.

**Author response:** This was a key expansion of the first comment. We added this consideration to our discussion regarding the impacts of citizen science on individuals’ idealized understanding of science versus their expectations when people engage in science directly as a practice (lines 539 – 541).

The text now says:

“For example, public engagement with the idealized concept of science (perhaps through media consumption) might differ from direct engagement with science in practice.”

6. Typo with regression beta coefficients in results.

**Author response:** Thank you for catching this error. We have corrected our reporting of unstandardized and standardized beta coefficients, adding clarifications in the main text, as well as figure and table captions (lines 343-368). In short, we report standardized beta in the text (as a measure of effect size to facilitate interpretation and comparisons) and we report unstandardized beta in the figures (in order to effectively calculate and display 95% confidence intervals).

**Addressing Journal Format Requirements:**

1. Style requirements and file naming.

**Author response:** We have reviewed style requirements and file names for accuracy.

2. Data availability and contact point.

**Author response:** We have removed the co-author as the contact point and instead included the link to our publicly available data in the Dryad repository. We have included the full citation and link to where our data can be located.

3. Inclusion of the full ethics statement in the “Methods” section.

**Author response:** We have included the full ethics statement, including the IRB protocol number and process for obtaining informed consent, in the main text of the methods (lines 229 – 231).

4. Higher resolution of figures 2, 3, and 4 needed.

**Author response:** We have uploaded higher resolution of figures, 2, 3, and 4.

5. Issues with copyrighted images in file S6_SurveyInstrument.pdf.

**Author response:** We have removed the NC State University logo that was causing copyright issues in the supplemental file of our questionnaire.

6. Inclusion of recommended citations from reviewers.

**Author response:** We have reviewed feedback. No additional citations were offered by reviewers.

7. Review of the reference list.

**Author response:** We have reviewed the reference list for completion and error. It is now accurate.
