# Supplementary material for: Americans’ multidimensional relationship with science: Is citizen science a path forward?
Source: PLoS One. 2026 Sep 11;21(9):e0357476. doi: 10.1371/journal.pone.0357476 (PMC13567779; doi:10.1371/journal.pone.0357476)
Supplement: Peer review history

# Peer review: Decision Letter 0

**DOI:** 10.1371/journal.pone.0357476.r001

**Authors:** Floris Vermeulen

31 May 2026

PONE-D-26-06030

Americans’ multidimensional relationship with science: how does citizen science fit in?

PLOS One?

Dear Dr. Joyner,

Thank you for submitting your manuscript to PLOS ONE. After careful consideration, we feel that it has merit but does not fully meet PLOS ONE’s publication criteria as it currently stands. Therefore, we invite you to submit a revised version of the manuscript that addresses the points raised during the review process.

Please submit your revised manuscript by Jul 15 2026 11:59PM. If you will need more time than this to complete your revisions, please reply to this message or contact the journal office at plosone@plos.org. When you're ready to submit your revision, log on to https://www.editorialmanager.com/pone/ and select the 'Submissions Needing Revision' folder to locate your manuscript file.

- A letter that responds to each point raised by the academic editor and reviewer(s). You should upload this letter as a separate file labeled 'Response to Reviewers'.
- A marked-up copy of your manuscript that highlights changes made to the original version. You should upload this as a separate file labeled 'Revised Manuscript with Track Changes'.
- An unmarked version of your revised paper without tracked changes. You should upload this as a separate file labeled 'Manuscript'.

If you would like to make changes to your financial disclosure, please include your updated statement in your cover letter. Guidelines for resubmitting your figure files are available below the reviewer comments at the end of this letter.

If applicable, we recommend that you deposit your laboratory protocols in protocols.io to enhance the reproducibility of your results. Protocols.io assigns your protocol its own identifier (DOI) so that it can be cited independently in the future. For instructions see: https://journals.plos.org/plosone/s/submission-guidelines#loc-laboratory-protocols. Additionally, PLOS ONE offers an option for publishing peer-reviewed Lab Protocol articles, which describe protocols hosted on protocols.io. Read more information on sharing protocols at https://plos.org/protocols?utm_medium=editorial-email&utm_source=authorletters&utm_campaign=protocols.

As the corresponding author, your ORCID iD is verified in the submission system and will appear in the published article. PLOS supports the use of ORCID, and we encourage all coauthors to register for an ORCID iD and use it as well. Please encourage your coauthors to verify their ORCID iD within the submission system before final acceptance, as unverified ORCID iDs will not appear in the published article. *Only*  the individual author can complete the verification step; PLOS staff *cannot*  verify ORCID iDs on behalf of authors.

We look forward to receiving your revised manuscript.

Kind regards,

Floris Vermeulen

Academic Editor

PLOS One

**Journal Requirements:**

When submitting your revision, we need you to address these additional requirements.

1. Please ensure that your manuscript meets PLOS ONE's style requirements, including those for file naming. The PLOS ONE style templates can be found at

https://journals.plos.org/plosone/s/file?id=wjVg/PLOSOne_formatting_sample_main_body.pdf and

https://journals.plos.org/plosone/s/file?id=ba62/PLOSOne_formatting_sample_title_authors_affiliations.pdf

2. In the online submission form you indicate that your data is not available for proprietary reasons and have provided a contact point for accessing this data. Please note that your current contact point is a co-author on this manuscript. According to our Data Policy, the contact point must not be an author on the manuscript and must be an institutional contact, ideally not an individual. Please revise your data statement to a non-author institutional point of contact, such as a data access or ethics committee, and send this to us via return email. Please also include contact information for the third party organization, and please include the full citation of where the data can be found.

3. Please include your full ethics statement in the ‘Methods’ section of your manuscript file. In your statement, please include the full name of the IRB or ethics committee who approved or waived your study, as well as whether or not you obtained informed written or verbal consent. If consent was waived for your study, please include this information in your statement as well.

4. Please upload a new copy of Figures 2, 3, and 4 as the detail is not clear. Please follow the link for more information:  https://journals.plos.org/plosone/s/figures

5. We note that [ S6_SurveyInstrument.pdf]  in your submission contain copyrighted images. All PLOS content is published under the Creative Commons Attribution License (CC BY 4.0), which means that the manuscript, images, and Supporting Information files will be freely available online, and any third party is permitted to access, download, copy, distribute, and use these materials in any way, even commercially, with proper attribution. For more information, see our copyright guidelines: http://journals.plos.org/plosone/s/licenses-and-copyright.

We require you to either (1) present written permission from the copyright holder to publish these figures specifically under the CC BY 4.0 license, or (2) remove the figures from your submission:

1. You may seek permission from the original copyright holder of Figure(s) [#] to publish the content specifically under the CC BY 4.0 license.

We recommend that you contact the original copyright holder with the Content Permission Form (http://journals.plos.org/plosone/s/file?id=7c09/content-permission-form.pdf) and the following text:

“I request permission for the open-access journal PLOS ONE to publish XXX under the Creative Commons Attribution License (CCAL) CC BY 4.0 (http://creativecommons.org/licenses/by/4.0/). Please be aware that this license allows unrestricted use and distribution, even commercially, by third parties. Please reply and provide explicit written permission to publish XXX under a CC BY license and complete the attached form.”

Please upload the completed Content Permission Form or other proof of granted permissions as an "Other" file with your submission.

In the figure caption of the copyrighted figure, please include the following text: “Reprinted from [ref] under a CC BY license, with permission from [name of publisher], original copyright [original copyright year].”

2. If you are unable to obtain permission from the original copyright holder to publish these figures under the CC BY 4.0 license or if the copyright holder’s requirements are incompatible with the CC BY 4.0 license, please either i) remove the figure or ii) supply a replacement figure that complies with the CC BY 4.0 license. Please check copyright information on all replacement figures and update the figure caption with source information. If applicable, please specify in the figure caption text when a figure is similar but not identical to the original image and is therefore for illustrative purposes only.

6. If the reviewer comments include a recommendation to cite specific previously published works, please review and evaluate these publications to determine whether they are relevant and should be cited. There is no requirement to cite these works unless the editor has indicated otherwise.

7. Please review your reference list to ensure that it is complete and correct. If you have cited papers that have been retracted, please include the rationale for doing so in the manuscript text, or remove these references and replace them with relevant current references. Any changes to the reference list should be mentioned in the rebuttal letter that accompanies your revised manuscript. If you need to cite a retracted article, indicate the article’s retracted status in the References list and also include a citation and full reference for the retraction notice.

[Note: HTML markup is below. Please do not edit.]

Reviewers' comments:

Reviewer's Responses to Questions

**Comments to the Author**

1. Is the manuscript technically sound, and do the data support the conclusions?

Reviewer #1: Yes

Reviewer #2: Yes

**********

2. Has the statistical analysis been performed appropriately and rigorously?

Reviewer #1: Yes

Reviewer #2: Yes

**********

3. Have the authors made all data underlying the findings in their manuscript fully available?

The PLOS Data policy

Reviewer #1: Yes

Reviewer #2: Yes

**********

4. Is the manuscript presented in an intelligible fashion and written in standard English?

Reviewer #1: Yes

Reviewer #2: Yes

**********

Reviewer #1: Thank you for the opportunity to review the manuscript entitled “Americans’ multidimensional relationship with science: how does citizen science fit in?”. The authors explore to what extent residents of the United States perceive science to be valuable and credible and how this is related to affinity for science. The authors clearly state why their research is relevant and provide detailed information on the methods that they have employed. In addition, the manuscript is well-structured.

Some remarks:

- Citizen science is a key concept in the manuscript, but the authors could expand on the definition of the concept. Citizen science is described as “a diverse methodology and discipline that can accommodate various research needs, facilitate broad engagement between public and scientific communities, and help foster positive relationships between science and society by influencing the science beliefs and science connections of individuals that participate”. However, this description is a bit vague and leaves the reader wondering what exactly can be seen as citizen science. For instance, the authors measure ‘participation in citizen science’ with a question asking respondents to select one of the following items ‘no’, ‘not sure’, ‘engaged as a leader’, ‘engaged as a participant’. Does the fact that these respondents participate in a Qualtrics panel or in this specific study imply that the answer should always be ‘yes’? Or is participating in a study not regarded as citizen science by the authors? In addition, because citizen science is a key concept it would be good to introduce a definition of the concept early on.

- The authors mention in the heading of Figure 3 what the reference categories are, but in the text it is unclear what groups are compared. For example, the authors find that “Black Americans were significantly less likely than other to report science has value to society”. Compared to what other group? All other ethnicity groups, or just the reference category of White?

- The authors could add effect sizes in their results section to indicate the impact of the significant effects that they have found.

Reviewer #2: The authors studies the relationship between the demographical factors, science beliefs, science connections, and citizen science for American residents. The theory, methods and results are developed well. Only a few revisions are needed to make the article clearer for the readers.

1. definition(1)

There seems to be room for discussion regarding what exactly science refers to in the definition of science, its mechanism explanation, and the design of the questionnaire: In the introduction to the definition of science, it seems to imply that there is an idealized tendency in the definition of science ("primarily rooted in positivist ideals of objectivity and 'truth claiming'"); when elaborating on how the public forms beliefs and connections with science, science seems to focus more on its practical aspect. However, there is likely to be a gap between the specific practice of science and the idealized science. The author's empirical results suggest that more exposure to or participation in science is related to lower perceived credibility, which seems to also imply this. In the questionnaire design, it seems that the authors did not reveal the definition of science written in the article to the participants. The authors could consider elaborating whether the three parts of the definition of science (definition, mechanism explanation, questionnaire design) can be aligned, for example, the authors might consider reducing the definition of science in this article to the practice of science to resolve the misunderstandings caused by the different emphases on the definition of science in different parts.

2. definition(2)

Furthermore, the authors also emphasizes the Western origin of science in the definition of science ("which encompasses the principles and methodologies of Western scientific practice"). The authors could consider adding an explanation regarding the necessity of mentioning the Western origin here. If necessary, additional explanation could be provided about the potential impact of this origin on the beliefs and connections of other non-Western groups towards science.

3.definition(3)

The term "STEM" may encompass different fields in different regions, such as in the UK and the US. There might be controversy over whether "social sciences" are included within this category. The authors may want to explain whether respondents implicitly have similar understandings regarding the term "STEM" in the theoretical framework and in the questionnaire design.

4. operationalisation

When measuring the concept of science identity, the authors used the phrase "Do you identify as a scientist?". The authors could consider reflecting on or elaborating on whether this measurement method is actually measuring the respondents' objective occupation, or whether it is measuring their subjective sense of identity despite their occupation.

5.results(1)

The explanations and discussions in the results regarding "more scientific engagement or participation is related to lower credibility" could consider including a discussion on the differences between science as an idealized vision and as actual practice.

6. results(2)

When reporting the results of the logistic regression model in the main text, it seems that the writing of B (raw estimate) and beta (standardized coefficient) does not match that in the table. It might be necessary to recheck the data reporting in the main text.

**********

what does this mean?). If published, this will include your full peer review and any attached files.

If you choose “no”, your identity will remain anonymous but your review may still be made public.

**Do you want your identity to be public for this peer review?**  For information about this choice, including consent withdrawal, please see our Privacy Policy

Reviewer #1: No

Reviewer #2: No

**********

[NOTE: If reviewer comments were submitted as an attachment file, they will be attached to this email and accessible via the submission site. Please log into your account, locate the manuscript record, and check for the action link "View Attachments". If this link does not appear, there are no attachment files.]

To ensure your figures meet our technical requirements, please review our figure guidelines: https://journals.plos.org/plosone/s/figures

You may also use PLOS’s free figure tool, NAAS, to help you prepare publication quality figures: https://journals.plos.org/plosone/s/figures#loc-tools-for-figure-preparation.

NAAS will assess whether your figures meet our technical requirements by comparing each figure against our figure specifications.

# Author comment: Author response to Decision Letter 1

**DOI:** 10.1371/journal.pone.0357476.r002

17 Aug 2026

Manuscript PONE-D-26-06030

Response to Reviewers

Dear Floris Vermeulen,

Thank you for the opportunity to submit a revised draft of our manuscript titled “Americans’ multidimensional relationship with science: Is citizen science a path forward?” for publication in PLOS One. We appreciate the time and effort that went into reviewing our manuscript and the thoughtful comments provided by reviewers to improve our paper. We have addressed all suggestions made by the reviewers and provide a point-by-point response below. All page numbers refer to the revised manuscript file with tracked changes. Additionally, we have addressed the formatting and data availability issues brought to our attention and summarized those changes at the end of this letter.

REVIEWERS COMMENTS TO THE AUTHORS:

REVIEWER 1

The authors clearly state why their research is relevant and provide detailed information on the methods that they have employed. In addition, the manuscript is well-structured.

Author response: Thank you for this feedback!

1. Comment regarding expansion on the definition of citizen science as a concept.

Author response: This was an excellent point that did relate to a point in our analysis of open-ended response data where some respondents interpreted their survey participation as a citizen science activity. As you suggested, we have added additional context to our definition of citizen science, specifically including criteria and additional citations for what activities constitute citizen science, including why survey research is not citizen science (lines 196 – 203).

The new Intro text says:

“Considering the broad engagement described above, we define citizen science participation as a process where members of public, including non-scientists, participate in one or more of the following scientific activities: identification of issues for study, experimental design, collection of data, data analyses, interpretation of study results, and communication of those results to others. Though the degree of public participation in a project may vary, an emphasis on public interest and impact beyond their participation as research subjects is a defining principle of citizen science [71]. Thus, participation in survey and clinical trial research is often excluded as citizen science practice [78].”

2. Clarification for what demographic group(s) were compared in the text supporting Figure 3: “Black Americans were significantly less likely than others to report science has value to society”.

Author response: Thank you for pointing this out; here we were comparing results between Black Americans to the reference category of White Americans. We have changed the text to read “Black Americans were significantly less likely to report that science has value to society (β = -0.149, p < 0.001) compared to White Americans” (line 347).

3. Possibly adding effect sizes to the results section to help interpret significant findings.

Author response: This was helpful to point out and did help us catch a typo in the reporting of these results. We updated the regression results in text to clarify that we were reporting relative effect sizes (standardized β) and updated the captions of fig 3 and fig 4 to clarify that the graphs represent unstandardized B coefficients in order to calculate 95% confidence intervals (lines 343 – 368). Researchers often use standardized beta to highlight relative effect sizes in regression models (Nieminen, 2022), [LL1.1]so this clarification should address the reviewer’s concern and facilitate interpretation of results, helping readers understand which predictor variables matter the most.

Nieminen, P. (2022). Application of standardized regression coefficient in meta-analysis. BioMedInformatics, 2(3), 434-458.

REVIEWER 2

The theory, methods and results are developed well. Only a few revisions are needed to make the article clearer for the readers.

Author response: Thank you for your feedback!

1. The definition of science, its mechanism explanation, and alignment with the questionnaire design.

Author response: After reviewing your feedback, we do agree that the multiple definitions of science are somewhat misaligned with how they are interpreted and operationalized, particularly between how we (the researchers) define science, how we believe the public might define science, and how we operationalize measures related to science in this work. We expanded our definition of science to address concept versus practice in our introduction (lines 87 – 96) and in the methods section describing the questionnaire design (239 – 243). In this later section, we also address how in our study the scope of interest was more in how individuals perceived their relationship with science, subjectively, rather than how they might define science. Ultimately, we wanted to people to respond to questions with their own view/definition of science in mind, not a formal definition prescribed by the researchers.[LL2.1]

Specifically, we added this explanation in Methods:

“Because our survey asked people to reflect on their personal relationship with science, we did not include a formal definition of “science” that could potentially limit how respondents interpreted and answered survey items about their science beliefs and connections. This allowed respondents to describe personal actions, feelings, and beliefs about science according to their own terms.”

2. Definition and mention of the Western origin of science.

Author response: This is a good point. We felt it was important to acknowledge the broader cultural paradigm that influences how people in the US might conceptualize science in concept and practice, as how they interpret science may influence their relationship with it. But (as pointed out by the reviewer) it’s important to acknowledge that people with non-western origins may have differing beliefs and connections with science compared to a majority of Americans, which we added in lines 93 – 96. A deeper investigation of these different cultural perceptions of science is beyond the scope of this study, however.[LL3.1]

Lines 93 – 96 now read:

“How people personally understand and define ‘science’ is also likely to vary across a diverse public encompassing people of varying demographic backgrounds (including non-Western origins), experiences, and access to scientific information.”

3. Definition and subjectivity of STEM

Author response: We agree that interpretations of STEM and what constitutes STEM may vary across respondents, though we tried to provide some clarity in our questionnaire by adding examples of STEM disciplines in parentheses (science, technology, engineering, or math[LL4.1]). We added a note on this in the limitations of our questionnaire design and discussed how that might also relate to the reviewers next comment about subjective science identity (lines 560 – 562).

4. Operationalization of science identity measure

Author response: This is an important clarification, and we have updated main text (line 253) and limitations (lines 560-562) to clarify that we are measuring a subjective sense of science identity that is not indicative of respondents’ self-described STEM occupation (and vice versa).

Lines 560-562 now say:

“Respondents' science identity and professional STEM affiliations are also subjective and more complex than our single-item measures could capture. As our study showed, for instance, STEM affiliation does not necessarily indicate science identity.”

5. Including discussion on the difference between idealized science and science as a practice in the results.

Author response: This was a key expansion of the first comment. We added this consideration to our discussion regarding the impacts of citizen science on individuals’ idealized understanding of science versus their expectations when people engage in science directly as a practice (lines 539 – 541).

The text now says:

“For example, public engagement with the idealized concept of science (perhaps through media consumption) might differ from direct engagement with science in practice.”

6. Typo with regression beta coefficients in results.

Author response: Thank you for catching this error. We have corrected our reporting of unstandardized and standardized beta coefficients, adding clarifications in the main text, as well as figure and table captions (lines 343-368). In short, we report standardized beta in the text (as a measure of effect size to facilitate interpretation and comparisons) and we report unstandardized beta in the figures (in order to effectively calculate and display 95% confidence intervals). [LL5.1]

ADDRESSING ADDITIONAL JOURNAL REQUIREMENTS:

1. Style requirements and file naming.

Author response: We have reviewed style requirements and file names for accuracy. [LL6.1]

2. Data availability and contact point.

Author response: We have removed the co-author as the contact point and instead included the link to our publicly available data in the Dryad repository. We have included the full citation and link to where our data can be located.

3. Inclusion of the full ethics statement in the “Methods” section.

Author response: We have included the full ethics statement, including the IRB protocol number and process for obtaining informed consent, in the main text of the methods (lines 229 – 231).

4. Higher resolution of figures 2, 3, and 4 needed.

Author response: We have uploaded higher resolution of figures, 2, 3, and 4.

5. Issues with copyrighted images in file S6_SurveyInstrument.pdf.

Author response: We have removed the NC State University logo that was causing copyright issues in the supplemental file of our questionnaire.

6. Inclusion of recommended citations from reviewers.

Author response: We have reviewed feedback. No additional citations were offered by reviewers.

7. Review of the reference list.

Author response: We have reviewed the reference list for completion and error. It is now accurate.

Attachment Submitted filename: Response to Reviewers.docx

# Editor report: Decision Letter 1

**DOI:** 10.1371/journal.pone.0357476.r003

**Authors:** Floris Vermeulen

18 Aug 2026

<p>Americans’ multidimensional relationship with science: Is citizen science a path forward?

PONE-D-26-06030R1

Dear Dr. Joyner,

We’re pleased to inform you that your manuscript has been judged scientifically suitable for publication and will be formally accepted for publication once it meets all outstanding technical requirements.

Within one week, you’ll receive an e-mail detailing the required amendments. When these have been addressed, you’ll receive a formal acceptance letter and your manuscript will be scheduled for publication.

An invoice will be generated when your article is formally accepted. Please note, if your institution has a publishing partnership with PLOS and your article meets the relevant criteria, all or part of your publication costs will be covered. Please make sure your user information is up-to-date by logging into Editorial Manager at Editorial Manager® and clicking the ‘Update My Information' link at the top of the page. For questions related to billing, please contact billing support.

If your institution or institutions have a press office, please notify them about your upcoming paper to help maximize its impact. If they’ll be preparing press materials, please inform our press team as soon as possible -- no later than 48 hours after receiving the formal acceptance. Your manuscript will remain under strict press embargo until 2 pm Eastern Time on the date of publication. For more information, please contact onepress@plos.org.

Kind regards,

Floris Vermeulen

Academic Editor

PLOS One

Additional Editor Comments (optional):

Reviewers' comments:

# Editor report: Acceptance letter

**DOI:** 10.1371/journal.pone.0357476.r004

**Authors:** Floris Vermeulen

PONE-D-26-06030R1

PLOS One

Dear Dr. Joyner,

I'm pleased to inform you that your manuscript has been deemed suitable for publication in PLOS One. Congratulations! Your manuscript is now being handed over to our production team.

At this stage, our production department will prepare your paper for publication. This includes ensuring the following:

* All references, tables, and figures are properly cited

* All relevant supporting information is included in the manuscript submission,

* There are no issues that prevent the paper from being properly typeset

You will receive further instructions from the production team, including instructions on how to review your proof when it is ready. Please keep in mind that we are working through a large volume of accepted articles, so please give us a few days to review your paper and let you know the next and final steps.

Lastly, if your institution or institutions have a press office, please let them know about your upcoming paper now to help maximize its impact. If they'll be preparing press materials, please inform our press team within the next 48 hours. Your manuscript will remain under strict press embargo until 2 pm Eastern Time on the date of publication. For more information, please contact onepress@plos.org.

You will receive an invoice from PLOS for your publication fee after your manuscript has reached the completed accept phase. If you receive an email requesting payment before acceptance or for any other service, this may be a phishing scheme. Learn how to identify phishing emails and protect your accounts at https://explore.plos.org/phishing.

If we can help with anything else, please email us at customercare@plos.org.

Thank you for submitting your work to PLOS One and supporting open access.

Kind regards,

PLOS One Editorial Office Staff

on behalf of

Dr. Floris Vermeulen

Academic Editor

PLOS One
